# Multi-omic biomarker identification and validation for diagnosing warzone-related post-traumatic stress disorder

**DOI:** 10.1038/s41380-019-0496-z

**Published:** 2019-09-10

**Authors:** Kelsey R. Dean, Rasha Hammamieh, Synthia H. Mellon, Duna Abu-Amara, Janine D. Flory, Guia Guffanti, Kai Wang, Bernie J. Daigle, Aarti Gautam, Inyoul Lee, Ruoting Yang, Lynn M. Almli, F. Saverio Bersani, Nabarun Chakraborty, Duncan Donohue, Kimberly Kerley, Taek-Kyun Kim, Eugene Laska, Min Young Lee, Daniel Lindqvist, Adriana Lori, Liangqun Lu, Burook Misganaw, Seid Muhie, Jennifer Newman, Nathan D. Price, Shizhen Qin, Victor I. Reus, Carole Siegel, Pramod R. Somvanshi, Gunjan S. Thakur, Yong Zhou, The PTSD Systems Biology Consortium, David Baxter, Linda Bierer, Esther Blessing, Ji Hoon Cho, Michelle Coy, Frank Desarnaud, Silvia Fossati, Allison Hoke, Raina Kumar, Meng Li, Iouri Makotkine, Stacy-Ann Miller, Linda Petzold, Laura Price, Meng Qian, Kelsey Scherler, Seshamalini Srinivasan, Anna Suessbrick, Li Tang, Xiaogang Wu, Gwyneth Wu, Changxin Wu, Leroy Hood, Kerry J. Ressler, Owen M. Wolkowitz, Rachel Yehuda, Marti Jett, Francis J. Doyle, Charles Marmar

**Affiliations:** 1 Department of Systems Biology, Harvard University, Cambridge, MA USA; 2 Harvard John A. Paulson School of Engineering and Applied Sciences, Harvard University, Cambridge, MA USA; 3 Integrative Systems Biology, US Army Medical Research and Materiel Command, USACEHR, Fort Detrick, Frederick, MD USA; 4 Department of Obstetrics, Gynecology, & Reproductive Sciences, University of California, San Francisco, CA USA; 5 Department of Psychiatry, New York Langone Medical School, New York, NY USA; 6 Department of Psychiatry, James J. Peters VA Medical Center, Bronx, NY USA; 7 Department of Psychiatry, Icahn School of Medicine at Mount Sinai, New York, NY USA; 8 Department of Psychiatry, McLean Hospital, Belmont, MA USA; 9 Institute for Systems Biology, Seattle, WA USA; 10 Departments of Biological Sciences and Computer Science, The University of Memphis, Memphis, TN USA; 11 Advanced Biomedical Computing Center, Frederick National Laboratory for Cancer Research, Frederick, MD USA; 12 Department of Psychiatry and Behavioral Sciences, Emory University, Atlanta, GA USA; 13 Department of Psychiatry, University of California, San Francisco, CA USA; 14 Department of Human Neurosciences, Sapienza University of Rome, Rome, Italy; 15 USACEHR, The Geneva Foundation, Frederick, MD USA; 16 Lund University, Faculty of Medicine, Department of Clinical Sciences, Lund, Sweden; 17 Department of Computer Science, University of California Santa Barbara, Santa Barbara, USA

**Keywords:** Psychiatric disorders, Diagnostic markers

## Abstract

Post-traumatic stress disorder (PTSD) impacts many veterans and active duty soldiers, but diagnosis can be problematic due to biases in self-disclosure of symptoms, stigma within military populations, and limitations identifying those at risk. Prior studies suggest that PTSD may be a systemic illness, affecting not just the brain, but the entire body. Therefore, disease signals likely span multiple biological domains, including genes, proteins, cells, tissues, and organism-level physiological changes. Identification of these signals could aid in diagnostics, treatment decision-making, and risk evaluation. In the search for PTSD diagnostic biomarkers, we ascertained over one million molecular, cellular, physiological, and clinical features from three cohorts of male veterans. In a discovery cohort of 83 warzone-related PTSD cases and 82 warzone-exposed controls, we identified a set of 343 candidate biomarkers. These candidate biomarkers were selected from an integrated approach using (1) data-driven methods, including Support Vector Machine with Recursive Feature Elimination and other standard or published methodologies, and (2) hypothesis-driven approaches, using previous genetic studies for polygenic risk, or other PTSD-related literature. After reassessment of ~30% of these participants, we refined this set of markers from 343 to 28, based on their performance and ability to track changes in phenotype over time. The final diagnostic panel of 28 features was validated in an independent cohort (26 cases, 26 controls) with good performance (AUC = 0.80, 81% accuracy, 85% sensitivity, and 77% specificity). The identification and validation of this diverse diagnostic panel represents a powerful and novel approach to improve accuracy and reduce bias in diagnosing combat-related PTSD.

## Introduction

Combat-related post-traumatic stress disorder (PTSD) has a lifetime prevalence of between 10.1%–30.9% in U.S. veterans of the Vietnam and subsequent conflicts, including the Iraq and Afghanistan wars [1–4]. PTSD is precipitated by experiencing or witnessing actual or threatened death, serious injury, or violence, and has symptoms that include re-experiencing, avoidance, negative thoughts, or moods associated with the traumatic event and hyperarousal (DSM-5 [5]). There is limited understanding of the biological processes underlying the core features of PTSD and associated psychiatric and somatic comorbidity [6].

Limited progress in the discovery of biological markers of PTSD has hampered accurate diagnosis, early identification of cases, staging and prognosis, stratification, personalized treatment, and new drug development. Additionally, individuals meeting diagnostic criteria for PTSD represent a heterogeneous group, as evidenced by differences in symptomatology, course, and treatment response [7]. Currently, case identification is limited by heavy reliance on self-reported symptoms for a disorder in which many trauma survivors under-report symptoms because of stigma, and some over-report symptoms for financial or other gains. Personalized treatment selection is limited by errors of omission (failing to identify individuals who would likely benefit from a specific behavioral or biological treatment) and errors of commission (treating individuals who are unlikely to benefit from a specific treatment), in part because of the lack of validated diagnostic and prognostic markers.

Previous PTSD biomarker studies have primarily focused on using gene expression for predicting risk and diagnosis [8–11]. These studies have demonstrated moderate success in identifying predictive and diagnostic markers, but have been limited due to small sample sizes, as well as the focus on an individual molecular data type. In cancer, multi-site, integrated multi-omic studies have shown great promise in generating novel insights into disease mechanism, diagnostic and predictive markers, and signals of progression and stratification [12–14]. These studies have included high-throughput ‘omics data such as genomics, transcriptomics, proteomics, methylomics, lipidomics and metabolomics [15]. By employing a systems biology framework, multi-omic datasests provide the ability to understand the underlying disease network-associated biological processes [16]. The systems biology approach aims to characterize a large and diverse set of molecules within an illness or individual by examining entire biological systems, not just individual components, allowing the assessment of interactions among levels of cellular pathology, ranging from DNA to circulating metabolites [17–19]. This approach has the potential to provide a more comprehensive characterization of illnesses, to track underlying biological dysregulation before clinical symptoms develop or worsen, to lead to the identification of improved diagnostic markers, and to allow for the discovery of novel targets for treatment [20].

In 2012, the Department of Defense initiated a multi-site “PTSD Systems Biology Consortium”, which applied multiple ‘omics technologies to the same sample of combat-exposed PTSD and control participants. The goals of the PTSD Systems Biology Consortium included developing a reproducible panel of blood-based biomarkers with good sensitivity and specificity for PTSD diagnosis. Here, we present identification and validation of a set of multi-omic biomarkers for diagnosing warzone-related PTSD.

## Materials and methods

### Study inclusion criteria

General inclusion criteria included being an Operation Enduring Freedom (OEF) and/or Operation Iraqi Freedom (OIF) male veteran between 20 and 60 years old, being able to understand the protocol and sign written informed consent, and meeting criteria for either PTSD-positive or PTSD-negative groups. PTSD-positive participants were defined as participants who met DSM-IV PTSD criteria for current warzone-related PTSD for at least 3 months duration, as indexed by the Clinician-Administered PTSD Scale (CAPS), with a minimum total score ≥ 40, which was calculated by summing each symptom on frequency and intensity ratings. Full criteria for DSM-IV diagnosis of PTSD was also met for all PTSD-positive participants. PTSD-negative controls were combat-exposed veterans that were negative for lifetime combat or civilian PTSD and had a current CAPS total score < 20. All study participants were exposed to DSM-IV PTSD Criterion A trauma during deployment. Detailed recruitment, enrollment, and exclusion criteria are listed in the Supplemental Material and Methods.

### Clinical assessment measures

The Structured Clinical Interview for DSM (SCID) was used to determine whether participants met DSM-IV diagnostic criteria for mood, anxiety, psychotic, and substance use disorders [21]. The CAPS was used to determine combat-related PTSD status, as well as the severity of current PTSD symptoms (past month is the “CAPS current”) and the severity of the most severe lifetime episode of combat-related PTSD (“CAPS lifetime”) [22].

### Molecular assays

Blood samples were assayed for many molecular species, including genetics, methylomics, proteomics, metabolomics, immune cell counts, cell aging, endocrine markers, microRNAs (miRNAs), cytokines, and more. DNA methylation was quantified using two approaches: a genome-wide unbiased approach, and a targeted sequencing-based approach. The genome-wide methylation approach quantified methylation using the Illumina Infinium HumanMethylation450K BeadChip array (Illumina Inc., CA). Using targets generated from this genome-wide approach, as well as other hypotheses generated from literature, a smaller set of methylation sites were evaluated by targeted sequencing via Zymo Research (Zymo Research, CA). Plasma miRNAs were evaluated using small RNA sequencing, and processed using sRNAnalyzer [23]. Proteins were evaluated using three methods: peptide quantification using selected reaction monitoring (SRM), quantification of six neurodegenerative disease-related markers using the Human Neurodegenerative Disease Panel 1, and quantification of serum levels of BDNF using a BDNF ELISA assay. Non-targeted metabolomics analysis was conducted using three platforms: ultrahigh performance liquid chromatography/tandem mass spectrometry (UHPLC/MS/MS^2^) optimized for basic species, UHPLC/MS/MS^2^ for acidic species, and gas chromatography/mass spectrometry (GC/MS). Additional data types, including routine clinical lab values and physiological measurements, were collected using standard procedures. Details on all molecular assays and blood draw information are contained in the Supplementary Materials (Table S2).

## Results

### Participant recruitment and multi-omic data generation

**Fig. 1:**
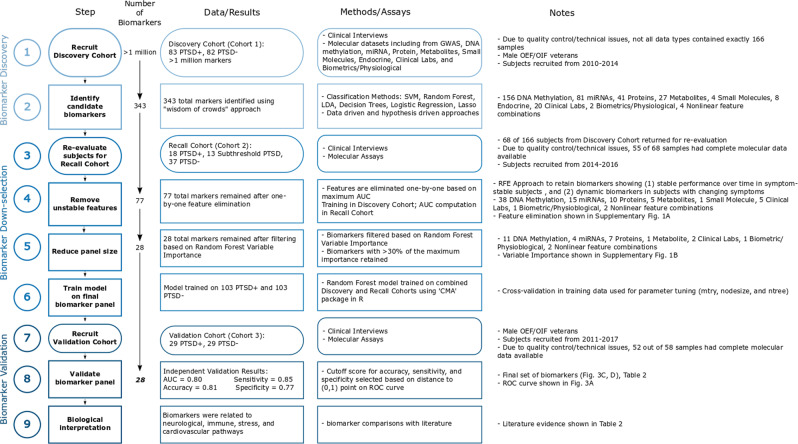
Overview of PTSD biomarker identification approach—details of cohort recruitment, and biomarker identification, down-selection, and validation

**Fig. 2:**
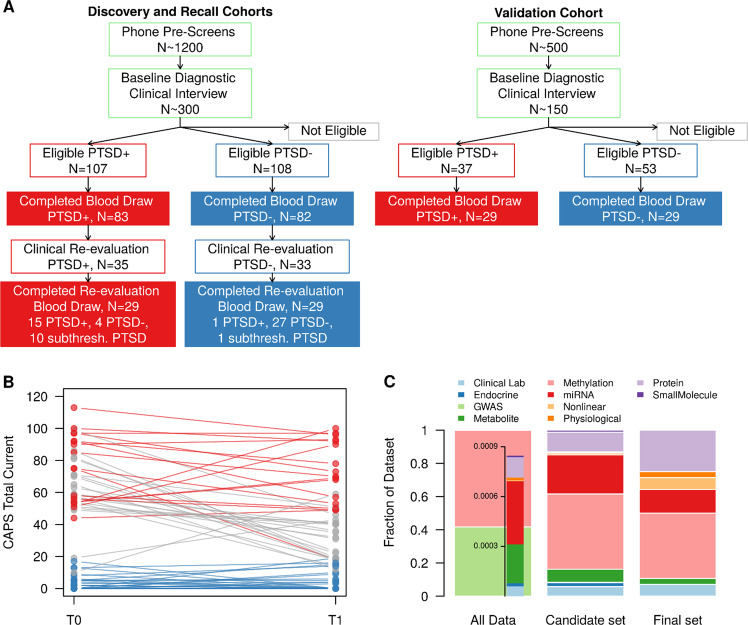
Overview of molecular datasets and cohort symptom severity. **a** Flow diagram for participant recruitment and enrollment. Participant eligibility was determined through a phone pre-screen and a baseline diagnostic clinical interview. Eligible participants completed fasting blood draws for multi-omic molecular assays. Participants in the initial discovery cohort were invited to return for follow-up in the recall cohort. Some participants returned with symptom changes, including “subthreshold” PTSD symptoms (below original study inclusion criteria). **b** Trajectory of PTSD symptoms in recalled participants. CAPS total for current symptoms at baseline (T0) and follow-up (T1) for each participant are connected. Participants who remained in the PTSD + group at both time points are shown in red. Participants who remained in the PTSD- group are shown blue. Participants with PTSD status changes are shown in gray, including participants who became “subthreshold” PTSD cases. **c** Distribution of molecular data types at three stages of biomarker identification: full exploratory dataset (All Data), reduced set of 343 potential biomarkers (candidate set) and the final panel of 28 biomarker (final set). Methylation and GWAS data represents 99% of initial data screen due to high-throughput arrays. Other molecular data types are well represented in the second and final stages of biomarker identification and selection

A set of three cohorts totaling 281 samples from male combat veterans from OEF/OIF conflicts were recruited as part of a larger study designed to identify biomarkers for PTSD diagnosis using a combination of clinical, genetic, endocrine, multi-omic, and imaging information (Fig. 1). Participants were recruited in three cohorts: discovery, recall, and validation (Fig. 2a and Table 1). The discovery cohort (cohort 1) consisted of 83 PTSD and 82 trauma-exposed control participants who met the inclusion and exclusion criteria (described in Materials and Methods and Supplementary Material). All participants completed clinical interviews and blood draws. After assessment of data quality, 77 PTSD and 74 trauma-exposed control samples were available with all completed blood marker assays. This discovery cohort was used to generate an initial pool of candidate biomarkers. Participants from the discovery cohort were invited back for clinical re-evaluation and a blood draw approximately three years after their initial evaluation. This cohort of recalled subjects (recall cohort, cohort 2), included 55 participants from the initial discovery cohort. Some of these participants showed PTSD symptom and status changes based on clinical assessment (Fig. 2b). In addition, some participants no longer met the original inclusion/exclusion criteria for the study; these participants had symptoms intermediate between the PTSD and control groups, in some cases meeting criteria for subthreshold PTSD. The 55 recall participants included 15 PTSD, 11 subthreshold PTSD, and 29 control participants. The third cohort, an independent group of 26 PTSD and 26 control participants, became the validation cohort (cohort 3), used for validating the final set of PTSD biomarkers.

**Table 1:** Summary of cohort demographics and clinical symptoms

	Cohort 1 (discovery cohort)		Cohort 2 (recalled cohort)			Cohort 3 (validation cohort)	
	PTSD + (*n* = 77)	PTSD− (*n* = 74)	PTSD + (*n* = 15)	Subthreshold PTSD (*n* = 11)	PTSD− (*n* = 29)	PTSD + (*n* = 26)	PTSD− (*n* = 26)
Age, years [mean (sd)]	32.8 (7.4)	32.6 (8.0)	33.7 (8.2)	35.6 (8.0)	36.6 (8.9)	36.8 (10.2)	33.0 (8.2)
Race/ethnicity [*n* (%)]							
Hispanic	34 (44%)	24 (32%)	9 (60%)	3 (27%)	12 (41%)	11 (42%)	2 (8%)
Non-Hispanic Asian	1 (1%)	5 (7%)	0 (0%)	0 (0%)	1 (3%)	3 (12%)	4 (15%)
Non-Hispanic black	21 (27%)	16 (22%)	4 (27%)	3 (27%)	5 (17%)	5 (19%)	4 (15%)
Non-Hispanic white	18 (23%)	24 (32%)	2 (13%)	4 (36%)	9 (31%)	7 (27%)	16 (62%)
Non-Hispanic other	3 (4%)	5 (7%)	0 (0%)	1 (9%)	2 (7%)	0 (0%)	0 (0%)
Education [*n* (%)]							
Less than 12th grade	2 (3%)	0 (0%)	1 (7%)	0 (0%)	0 (0%)	1 (4%)	0 (0%)
HS diploma or GED	27 (35%)	13 (18%)	2 (13%)	1 (9%)	4 (14%)	10 (38%)	6 (23%)
2 years college, AA degree	23 (30%)	21 (28%)	6 (40%)	4 (36%)	3 (10%)	7 (27%)	6 (23%)
4 years college, BA degree	22 (29%)	28 (38%)	5 (33%)	4 (36%)	19 (66%)	5 (19%)	7 (27%)
Master’s degree	3 (4%)	11 (15%)	1 (7%)	2 (18%)	3 (10%)	3 (12%)	7 (27%)
Doctoral degree	0 (0%)	1 (1%)	0 (0%)	0 (0%)	0 (0%)	0 (0%)	0 (0%)
Body mass index [mean (sd)]	30.1 (5.1)	28.1 (4.3)	32.2 (5.3)	31.1 (8.0)	27.2 (6.7)	29.4 (6.7)	26.9 (2.5)
Cholesterol [mean (sd)]							
HDL cholesterol	47.6 (11.9)	50.1 (13.3)	43.0 (8.0)	45.9 (11.1)	53.3 (13.9)	47.3 (12.8)	53.3 (11.9)
LDL cholesterol	108.2 (32.3)	100.0 (25.4)	115.1 (26.4)	110.3 (37.7)	105.3 (27.6)	116.2 (30.0)	98.1 (31.2)
HbA1c [mean (sd)]	5.4 (0.9)	5.4 (0.4)	5.6 (0.8)	5.6 (0.7)	5.3 (0.5)	5.4 (0.3)	5.3 (0.4)
PTSD Severity, Total CAPS [mean (sd)]	68.0 (16.1)	3.6 (4.9)	69.5 (18.5)	37.6 (9.0)	5.2 (7.3)	67.2 (19.4)	2.7 (4.5)
Early trauma exposure, ETISR total [mean (sd)]	7.7 (5.8)	5.1 (4.0)	5.2 (4.6)	6.9 (4.8)	4.5 (3.9)	7.2 (4.8)	4.9 (3.3)
Major depressive disorder [*n* (%)]	42 (55%)	0 (0%)	9 (64%)	2 (18%)	0 (0%)	9 (35%)	1 (4%)
Peritraumatic dissociate experience, Rater version [mean (sd)]	1.8 (0.4)	1.2 (0.2)	1.8 (0.5)	1.8 (0.5)	1.3 (0.3)	1.8 (0.4)	1.2 (0.2)
Peritraumatic distress inventory, Rater version [mean (sd)]	2.1 (0.8)	1.1 (0.6)	2.2 (0.7)	2.1 (0.9)	1.0 (0.6)	2.0 (0.9)	1.0 (0.7)
Sleep quality, PSQI [mean (sd)]	13.1 (3.3)	5.9 (3.6)	11.8 (3.5)	10.7 (4.2)	5.6 (3.0)	11.9 (3.6)	6.2 (3.3)
Number of tours [mean (sd)]	1.8 (0.9)	1.7 (0.8)	1.5 (0.6)	1.6 (0.7)	1.9 (1.1)	1.3 (0.6)	2.1 (1.5)

### PTSD cohorts and multi-omic datasets

To identify a minimally invasive PTSD diagnostic panel, blood-based multi-omics and other analytes were assayed for each individual (and during both visits for recalled participants), including DNA methylation, proteomics, metabolomics, miRNAs, small molecules, endocrine markers, and routine clinical lab panels. Additionally, physiological measures were recorded and nonlinear marker combinations were computed. Using a strategy described in the next sections, a robust and diverse 28-member biomarker panel for diagnosing PTSD was identified from this pool of more than one million markers (Fig. 2c).

### Three-stage biomarker identification and down-selection from exploratory set of multi-omic data

We used a “wisdom of crowds” approach to identify candidate PTSD biomarkers from the large set of measured blood analytes. Utilizing domain area expertize of multiple researchers, as well as multiple algorithms and methodologies, collective intelligence has the potential to identify successful candidate biomarkers from a large dataset, particularly when knowledge is limited. Collective intelligence and “wisdom of crowds” approaches are often used in financial modeling and predictions [24], have been evaluated in medical decision-making [25], and are the motivation for ensemble classification methods, which have been shown to outperform individual classifiers [26].

From a diverse set of data-driven, hypothesis-driven, hybrid, and other approaches (Table S3), we identified a set of candidate diagnostic panels, totaling 343 unique potential biomarkers (Step 2 from Fig. 1 and Table S4). These approaches included COMBINER [27], polygenic risk [28, 29], as well as traditional Support Vector Machine with Recursive Feature Elimination (SVM-RFE), random forest, and other classification algorithms, and feature selection approaches, including *p*-value, *q*-value, and fold-change filtering. Details of these algorithms are listed in the Supplementary Material. To filter and refine the pool of candidate biomarkers, we used data from recalled participants (recall cohort, cohort 2). Many of these returning participants experienced symptom changes over the 3.3 ± 0.9 years (mean ± sd) between the initial and follow-up evaluation. CAPS totals for recalled participants at both time points are shown in Fig. 2b. The panel was refined using the recall cohort along with a two-stage down-selection approach to select the final set of PTSD biomarkers (Steps 4–5 from Fig. 1).

The two-stage down-selection process is based on the following methodology. In the first stage, poor performing candidate biomarkers were removed one-by-one based on the largest average AUC of the remaining biomarker set (Step 4, Fig. 1). The trajectory of AUC scores in the recall cohort is shown in Supplementary Fig. 1A, showing the average AUC at each step of the one-by-one elimination. The biomarker set with the largest average AUC prior to the final performance decline was selected, resulting in 77 remaining biomarkers.

To further reduce the number of features in the panel, we implemented a second stage of down-selection, based on random forest variable importance (Fig. 1, Step 5). Using the recall cohort, the remaining 77 biomarkers were sorted based on random forest variable importance (Supplementary Fig. 1B). We retained biomarkers with importance >30% of the maximum importance score for the final biomarker panel (*n* = 28). The dynamics and distribution of these 28 biomarkers in the discovery and recall cohorts is shown in Supplementary Figs. 2 and 3.

### Validation of a robust, multi-omic PTSD biomarker panel

**Fig. 3:**
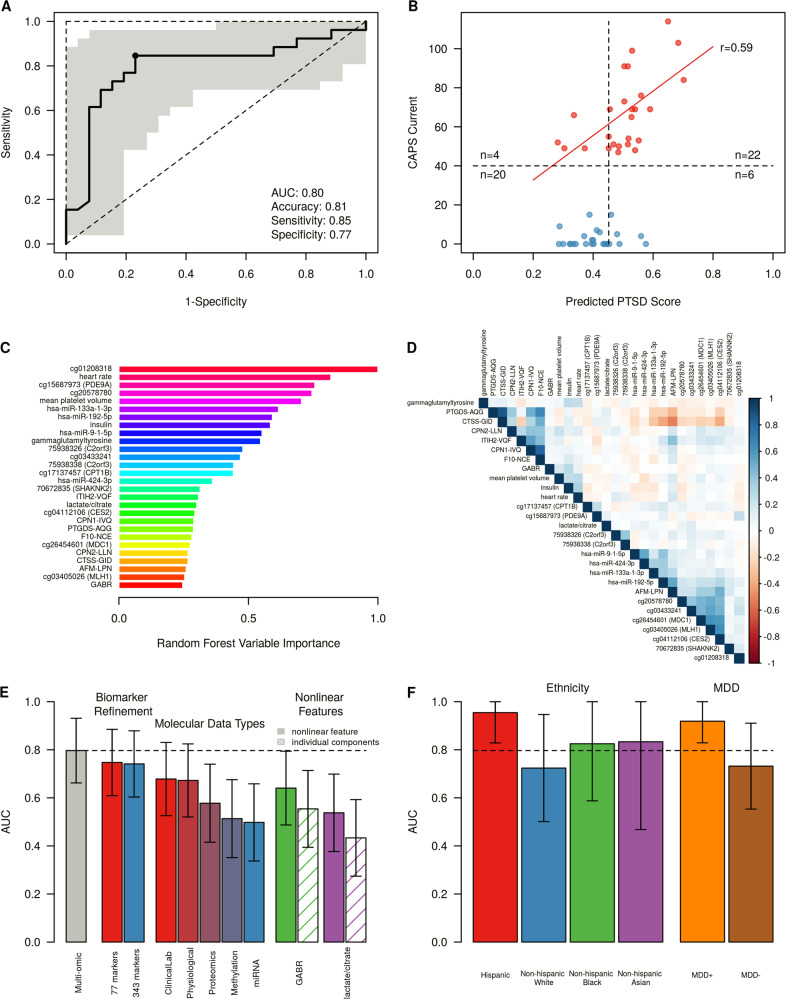
Validation of biomarker panels. **a** ROC curve for identified biomarker panel (28 markers), illustrating good performance in an independent validation dataset (26 cases, 26 controls). Shaded region indicates 95% confidence interval, determined by 2000 bootstrapping iterations. Operating point closest to (0,1) on ROC curve used for calculating sensitivity, specificity, and accuracy. **b** Predicted probability of PTSD based on trained random forest model using a biomarker panel of 28 features. In PTSD participants, predicted PTSD probability is correlated with PTSD symptom severity, measured by CAPS (*r* = 0.59, *p* < 0.01). **c** Random forest variable importance of the final 28 biomarkers. Variable importance was determined using biomarker model training data (cohorts 1 and 2). The top 10 biomarkers, based on random forest variable importance, contain multiple data types, including methylation markers (cg01208318, cg20578780, and cg15687973), physiological features (heart rate), miRNAs (miR-133a-1-3p, miR-192-5p, and miR-9-1-5p), clinical lab measurements (insulin and mean platelet volume), and metabolites (gammaglutamyltyrosine). **d** Correlation between PTSD biomarkers. Pearson correlation coefficients were computed in the combined set of all three cohorts. The final set of identified biomarkers show small clusters of moderately correlated features, primarily grouped by molecular data type (proteins, miRNAs, and methylation markers). **e** Biomarker panel performance evaluation during panel refinement, across molecular data types, and in nonlinear features. The validation AUC improves after biomarker down-selection and model refinement. The final biomarker panel validates with greater AUC over the initial biomarker candidate pool (343 markers, AUC = 0.74), and stage one refined panel (77 markers, AUC = 0.75). The final multi-omic panel also outperforms each individual molecular data type. Performance metrics for nonlinear feature combinations, Global Arginine Bioavailability Ratio (GABR) and lactate/citrate. Both nonlinear combinations outperform their individual components in AUC (0.60 vs. 0.51 and 0.55 vs. 0.52 in GABR and lactate/citrate, respectively). Error bars indicate 95% confidence interval, determined by 2000 bootstrapping iterations. **f** Validation performance by ethnicity, and in the presence of major depressive disorder (MDD). Validation performance in Hispanic participants was higher than other ethnicities (non-Hispanic White, non-Hispanic Black, non-Hispanic Asian). PTSD cases with comorbid MDD (*n* = 9) are easily distinguishable from all combat-exposed controls (*n* = 26), with AUC = 0.92, while PTSD cases without comorbid MDD (*n* = 17) are only moderately distinguishable from controls (*n* = 26), with AUC = 0.73

After the two-stage feature reduction strategy, the final biomarker set consisted of 28 features, including methylation, metabolomics, miRNA, protein, and other data types. A random forest model trained on the combined cohorts 1 and 2 predicted PTSD status in an independent validation set (cohort 3) with an area under the ROC curve (AUC) of 0.80 (95% CI 0.66–0.93, Fig. 3a). Using the point closest to (0,1) on the ROC curve (shown in Fig. 3a), the model was validated with an accuracy of 81%, sensitivity of 85%, and specificity of 77%. The PTSD participants in the validation cohort had CAPS scores ranging from 47–114. We found that predicted PTSD scores from the random forest model for these cases were correlated with total CAPS (*r* = 0.59, *p* = 0.001), indicating the current biomarker model predicts not only disease status, but potentially PTSD symptom severity of cases (Fig. 3b). In addition, predicted PTSD scores were moderately correlated with DSM-IV re-experiencing, avoidance, and hyperarousal symptoms (*r* = 0.44–0.53, Supplementary Fig. 4), suggesting that the identified molecular markers are not specific to a single symptom cluster, but to overall symptoms.

Overall, the set of identified PTSD biomarkers contains many molecular data types (DNA methylation, miRNAs, proteins, metabolites, and others), with signals primarily including under-expressed proteins and miRNAs, and signatures of both DNA hyper- and hypomethylation. Of the 28 markers comprising the final panel, 16 markers had consistent fold-change directions in all three cohorts (Table 2). Five of the final 28 markers were retained during panel refinement even though the fold-change direction was inconsistent between the discovery and recall cohorts, indicating that these features may contain relevant PTSD signal that is not purely measured by group differences in mean. A post hoc analysis of the biomarker panel performance without these inconsistent features resulted in decreased validation performance (AUC = 0.74 and 0.71 when using only markers with consistent fold-change directions across the discovery and recall cohorts (23 markers), and all three cohorts (16 markers), respectively).

**Table 2:** Overview of biomarker signals in each of the three cohorts

		Cohort 1 (Discovery Cohort)	Cohort 2 (Recalled Cohort)	Cohort 3 (Validation Cohort)	Findings from the literature
70672835 (SHANK2)	Methylation	↓	↑	↓	Genetic variants of SHANK2 associated with schizophrenia [30]
75938326 (C2orf3)	Methylation	↑	↑	↑	
75938338 (C2orf3)	Methylation	↑	↑	↑	
AFM-LPN	Protein	↑	↓	↓	
cg01208318	Methylation	↓	↓	↓	
cg03405026 (MLH1)	Methylation	↑	↓	↓	
cg03433241	Methylation	↑	↑	↓	
cg04112106 (CES2)	Methylation	↑	↑	↓	
cg15687973 (PDE9A)	Methylation	↓	↓	↑	PDE9A expression has been associated with monoamine neurotransmitter regulation and depression [31]
cg17137457 (CPT1B)	Methylation	↓	↓	↓	CPT1B expression has been associated with rodent stress and human PTSD [32]
cg20578780	Methylation	↓	↓	↓	
cg26454601 (MDC1)	Methylation	↑	↑	↓	
CPN1-IVQ	Protein	↓	↓	↓	
CPN2-LLN	Protein	↓	↑	↑	
CTSS-GID	Protein	↑	↑	↓	
F10-NCE	Protein	↑	↓	↑	Decreased coagulation in PTSD [10]
Global Arginine Bioavailability Ratio (GABR)	Metabolite	↓	↑	↑	GABR is decreased in patients with MDD [33]; previous decreased PTSD finding published in [34]
Gammaglutamyltyrosine	Metabolite	↑	↑	↑	Gammaglutamyltyrosine negatively correlated with leukocyte telomere length [35]
heart rate	Physiological	↑	↑	↑	Elevated heart rate following trauma associated with development of PTSD [36]
hsa-miR-133a-3p	miRNA	↓	↓	↓	
hsa-miR-192-5p	miRNA	↓	↓	↑	Abundant in liver; associated with obesity and diabetes [37,38]
hsa-miR-424-3p	miRNA	↓	↓	↓	miR-424-3p has been associated with inflammation [39]
hsa-miR-9-5p	miRNA	↓	↓	↓	Enriched in brain tissue and regulates neurogenesis [40]
Insulin	Clinical Labs	↑	↑	↑	Increased insulin resistance in veterans with PTSD [41]
ITIH2-VQF	Protein	↓	↓	↓	
Lactate/citrate	Metabolite	↑	↑	↑	Lactate has been considered panic-inducing in both Panic Disorder and PTSD [42]
Mean platelet volume	Clinical Labs	↑	↑	↑	Increased mean platelet volume associated with panic disorder [43] and major depression [44]
PTGDS-AQG	Protein	↓	↓	↓	Prostaglandin dysregulation has been associated with both rodent stress models of PTSD and in pathways related to human PTSD [45, 46]

Arrows indicate upregulated and downregulated signals, respectively. Underlined arrows indicate consistent signals across all three cohorts, while non-underlined arrows indicate contradictory signal directions

Using random forest variable importance, the top 10 biomarkers from the final 28-marker panel included five of the six molecular data types: DNA methylation, physiological, miRNAs, clinical lab measures, and metabolites (Fig. 3c). These data types contribute primarily uncorrelated signals, with only small clusters of moderate to highly correlated biomarkers from three data types: proteins, miRNAs, and DNA methylation (Fig. 3d).

Through the biomarker identification and down-selection process, two intermediate biomarker sets were identified, consisting of 343 and 77 candidate biomarkers. Trained random forest models on these biomarker sets validated with slightly lower AUCs than the final biomarker panel (AUCs of 0.74, 0.75, and 0.80 in the 343, 77, and 28 biomarker panels; Fig. 3e). The consistent validation AUC indicates robust signal in these sets of candidate biomarkers, without loss of signal during down-selection from 343 to 28 features. The final panel of 28 markers consisted of six different data types: routine clinical lab markers, metabolites, DNA methylation marks, miRNAs, proteins, and physiological measurements. The combined panel out-performed all six panels composed of each individual data type (Fig. 3e), demonstrating the power of combining different types of markers in a diverse biomarker panel, capable of capturing the complexities of PTSD.

Two biomarker features included in our final panel are computed, nonlinear metrics: Global Arginine Bioavailability Ratio (GABR, defined as arginine/[ornithine + citrulline]) and lactate/citrate. These computed ratios outperform their combined individual components in predictive performance, indicating biologically-driven nonlinear features may enhance low signals (Fig. 3e). In addition, these ratios begin to alleviate single-sample normalization issues that need to be addressed for clinical use of a biomarker panel.

### Evaluation of clinical and demographic factors

The cohorts recruited for this study are diverse in terms of ethnicity, educational background, clinical symptoms, overall health, and comorbid diseases and conditions. The heterogeneity of the participants included in these three cohorts, including race, age, and clinical comorbidities, as well as PTSD severity are shown in Table 1. To evaluate the performance of this biomarker panel in the context of participant demographics and other clinical factors, we computed biomarker performance in stratified subsets of the validation cohort. While biomarker performance was highest in Hispanic participants (AUC = 0.95), we observed no statistically significant differences in AUC across ethnicities (Fig. 3f). Multiple studies have examined the increased prevalence and greater symptom severity of PTSD in Hispanic populations [47, 48], which may correspond to stronger biological signals, leading to the differences in AUC.

In the validation cohort, 35% of PTSD cases also met the criteria for major depressive disorder (MDD). Using the identified biomarker panel and model, these PTSD + /MDD + cases could be distinguished from all controls with an AUC of 0.92, while the PTSD + /MDD – could only be distinguished from controls with an AUC of 0.73 (Fig. 3f). Similarly, predicted PTSD scores were more strongly correlated with PTSD symptom severity in PTSD + /MDD + participants than in PTSD + /MDD– participants, with *r* = 0.64 and *r* = 0.37, respectively (Supplementary Fig. 5). This decrease in prediction accuracy and correlation with PTSD symptoms in the absence of comorbid MDD indicates a potential overlap of biological signals for MDD and PTSD that should be explored further.

## Discussion

This study presents the identification and validation of a biomarker panel for the diagnosis of combat-related PTSD. The panel consists of 28 features that perform well in identifying PTSD cases from combat-exposed controls in a male, veteran population (81% accuracy). Some of the biomarkers have been linked to PTSD previously, including elevated heart rate [36] and decreased level of coagulation factors [10], and other included markers have been linked to MDD, anxiety, and other comorbid conditions, including platelet volume [43, 44], insulin resistance [41, 49], alterations in the SHANK2 gene [30], and PDE9A expression [31] (Table 2).

In particular, the circulating miRNAs selected in the panel reflect the diverse pathology and comorbidities present in PTSD populations, including connections to metabolic diseases and cardiovascular conditions. The miR-133-3p, a member of myomiRs that are highly abundant in muscle, including cardiac muscle, has been implicated in cardiomyocyte differentiation and proliferation [50]. The circulating miR-133-3p level has been linked to various cardiovascular disorders, including myocardial infarction, heart failure, and cardiac fibrosis [51, 52]. The miR-9-5p is enriched in brain [40] and known as a regulator for neurogenesis. It is also involved in heart development and heart hypertrophy [53]. The miR-192 is highly abundant in the liver and circulating miR-192-5p levels have been associated with various liver conditions as well metabolic diseases such as obesity and diabetes [37, 38]. The circulating miR-192 level has also been used as a biomarker for ischemic heart failure [54].

In addition to molecular markers, our approach selected heart rate as a contributor to the PTSD diagnostic panel. More than two decades ago, heart rate differences were observed between eventual PTSD cases and controls during emergency room visits and at 1-week follow-ups after trauma [36]. While these differences did not persist for longer time points in Shalev’s study, we observed significant mean group differences for heart rates in two of the three cohorts from this study, a number of years following trauma exposure (*p* < 0.01 for discovery and validation cohorts). Heart rate alone predicts diagnosis of PTSD in the validation cohort with 69% accuracy. Of note, removing heart rate from our biomarker panel did not result in significantly decreased model performance (molecular-only panel without heart rate still achieves 75% accuracy).

Following the heart rate analysis, we evaluated all other biomarkers contained in the panel individually. Three other markers achieved at least 60% accuracy in the validation cohort: gammaglutamyltyrosine, insulin, and cg01208318. However, using any of these markers individually resulted in greater variance in validation accuracy, based on 2000 bootstrapping iterations. Additionally, we note that the most important markers selected during model refinement (based on Random Forest Variable Importance, Supplementary Fig. 1B), were not the top-performing individual markers in the validation cohort. Without an additional validation cohort, validation performance cannot be used to hand-select top-performing individual markers. During additional rounds of panel validation and development, individual markers and smaller subsets of this biomarker panel should be evaluated.

### Strengths and limitations

The cohorts recruited for this study were subject to strict inclusion and exclusion criteria, intentionally creating a pool of moderate to severe cases of combat-related PTSD to compare with asymptomatic controls among men deployed to Iraq and/or Afghanistan. To understand the clinical utility of the proposed biomarker panel, further validation is required in other PTSD populations, including active duty soldiers, populations with civilian trauma, female cohorts, and carefully phenotyped populations with and without many conditions commonly comorbid with PTSD. This study design may have allowed for the clearest and strongest signals of combat-related PTSD to emerge, but will need additional validation in cohorts of individuals with chronic PTSD (>10 years), individuals who recover from PTSD, and those with intermediate PTSD symptoms (CAPS from 20–40), where the current model performance may be decreased. Additionally, this study used DSM-IV criteria for diagnosing PTSD to ensure consistency across all cohorts. Hoge et al. [55] determined that 30% of combat veterans who meet DSM-IV diagnostic criteria for PTSD do not meet DSM-5 criteria for PTSD. The impact of using DSM-5 should be evaluated for this specific set of biomarkers in future cohorts.

Many studies have emphasized the high rates of PTSD comorbidity with other conditions, including depression [56], anxiety [57], alcoholism and substance abuse [58], cardiovascular disease [59], diabetes [60], and others. A robust PTSD biomarker panel should be (i) specific to PTSD and not any of these or other comorbidities, and (ii) able to detect PTSD in both the presence and absence of these comorbid conditions. To further identify potential confounders, additional samples including MDD without PTSD, diabetes with and without PTSD, and other conditions should be studied to evaluate the specificity of the panel further.

In an exploratory search of more than one million markers, we assayed a range of molecular data types, including DNA methylation marks, proteins, miRNAs, and metabolites. Owing to quality control and other limitations, several molecular data types were incomplete and therefore excluded from biomarker identification and refinement. These included gene expression, immune cell counts, and cytokine assays. Some of these assays were completed for the discovery cohort, and were included in early approaches for candidate biomarker selection. Any identified biomarker candidates from these assays were removed prior to down-selection and validation due to lack of data in recall and validation cohorts. The presence of these markers in the discovery phase may have influenced the selection of candidate biomarkers for some of the machine learning approaches. However, the exclusion of these datasets was not based on biomarker validation performance and therefore could not have affected the final accuracy and performance of the 28-marker panel.

In summary, we have presented a robust multi-omic panel for predicting combat-related PTSD diagnosis in male veteran populations. These 28 biomarkers include features from DNA methylation, proteins, miRNAs, metabolites, and other molecular and physiological measurements. In an independent validation cohort, we predicted PTSD diagnosis with 81% accuracy, 85% sensitivity, and 77% specificity, indicating a blood-based screening or diagnostic tool is promising for identifying PTSD, particularly in males with warzone-related PTSD.

### Disclaimer

The views, opinions and/or findings contained in this report are those of the authors and should not be construed as an official Department of the Army position, policy or decision, unless so designated by other official documentation. The views and conclusions contained in this document are those of the authors and should not be interpreted as representing the official policies, either expressed or implied, of the Army Research Laboratory or the U.S. Government. The U.S. Government is authorized to reproduce and distribute reprints for Government purposes notwithstanding any copyright notation herein.

## Supplementary information

Supplemental Materials and Methods

## Data Availability

Molecular, clinical, and demographic datasets for all three cohorts are available through the SysBioCube [61], at https://sysbiocube-abcc.ncifcrf.gov.
