# Supplementary material for: Multi-omic biomarker identification and validation for diagnosing warzone-related post-traumatic stress disorder
Source: Mol Psychiatry. 2019 Sep 10;25(12):3337–49. doi: 10.1038/s41380-019-0496-z (PMC7714692; doi:10.1038/s41380-019-0496-z)
Supplement: Supplementary file 1 — Supplemental Materials and Methods [file 41380_2019_496_MOESM1_ESM.docx]

**Supplemental Materials and Methods**

Table S1. Additional Consortium Members.

| Name | Affiliation |
| --- | --- |
| David Baxter | Institute for Systems Biology, Seattle, WA |
| Linda Bierer | Department of Psychiatry, James J. Peters VA Medical Center, Bronx, NY |
| Esther Blessing | Department of Psychiatry, New York Langone Medical School, NY, NY |
| Ji Hoon Cho | Institute for Systems Biology, Seattle, WA |
| Michelle Coy | Department of Psychiatry, University of California, San Francisco |
| Frank Desarnaud | Department of Psychiatry, James J. Peters VA Medical Center, Bronx, NY |
| Silvia Fossati | Department of Psychiatry, New York Langone Medical School, NY, NY |
| Allison Hoke | USACEHR, The Geneva Foundation, Frederick, MD |
| Raina Kumar | Advanced Biomedical Computing Center, Frederick National Laboratory for Cancer Research, Frederick, MD |
| Meng Li | Department of Psychiatry, New York Langone Medical School, NY, NY |
| Iouri Makotkine | Department of Psychiatry, James J. Peters VA Medical Center, Bronx, NY |
| Stacy-Ann Miller | USACEHR, The Geneva Foundation, Frederick, MD |
| Linda Petzold | Department of Computer Science, University of California Santa Barbara |
| Laura Price | Department of Psychiatry, New York Langone Medical School, NY, NY |
| Meng Qian | Department of Psychiatry, New York Langone Medical School, NY, NY |
| Kelsey Scherler | Institute for Systems Biology, Seattle, WA |
| Seshamalini Srinivasan | USACEHR, The Geneva Foundation, Frederick, MD |
| Anna Suessbrick | Department of Psychiatry, New York Langone Medical School, NY, NY |
| Li Tang | Institute for Systems Biology, Seattle, WA |
| Xiaogang Wu | Institute for Systems Biology, Seattle, WA |
| Gwyneth Wu | Department of Psychiatry, University of California, San Francisco |
| Changxin Wu | Department of Psychiatry, James J. Peters VA Medical Center, Bronx, NY |

Cohort Recruitment, Exclusion Criteria, and Blood Draws

Participants were recruited from the Mental Health Services of the Manhattan, Bronx and Brooklyn Veterans Affairs Medical Centers, other regional VA medical centers, Veterans Service Organizations, National Guard, reservist agencies and organizations from the general community. Recruitment methods included flyers, in-person presentations, media advertisements, internet postings (e.g. Craigslist) and referral from clinicians.

Participants were compensated for their participation. Each enrolled participant completed an initial phone screen to determine preliminary eligibility. Based on phone screen eligibility, participants were then invited for an in-person informed consent process, which included a complete study description. Participants that consented then completed a clinical assessment using the Clinician Administered PTSD Scale (CAPS) ^14^ and Structured Clinical Interview for DSM-IV (SCID) ^15^, to determine eligibility for the study. The CAPS was used to determine the presence versus absence of a DSM-IV diagnosis of PTSD related to deployment trauma exposure and to assess symptom severity of the combat-related PTSD. The SCID was conducted to assess for mood, anxiety, psychosis, and substance related disorders. The SCID PTSD module was also used to determine whether participants had ever met DSM-IV diagnostic criteria for PTSD related to a non-deployment traumatic exposure.

Exclusion criteria included history of open head injury or closed head injury with loss of consciousness of more than 10 min, any head injury leading to a currently elevated score of post concussive symptoms, current drug abuse or dependence within the past year, current alcohol dependence or history of dependence within the past 8 months, lifetime history of any psychiatric disorder with psychotic features, bipolar disorder, or obsessive-compulsive disorder, currently exposed to recurrent trauma or exposed to a traumatic event within the past 3 months, participants with prominent suicidal or homicidal ideation, neurologic disorder or systemic illness affecting central nervous system function, anemia, recent blood donation in the past 2 months, and participants who were not stable for 2+ months on psychiatric medication, anticonvulsants, antihypertensive medication or sympathomimetic medication.

All clinicians who conducted the clinical interviews for this study were post-doctorate level psychologists who had several years of experience working with veterans and trauma survivors. All PTSD diagnoses were calibrated weekly with the PTSD program clinical team across sites, including a senior clinician, which ensured a similar use of diagnostic measures and consistent application of inclusion and exclusion criteria.

In addition, participants completed self-report questionnaires, which provide background information as well as assessments related to PTSD symptoms, head injuries, physical health, depression, anxiety, alcohol use, trauma exposure, and measures of potential covariates for use in biomarkers analyses.

Participants reported to the laboratory at James J. Peters VA Medical Center (JJP VAMC) or Icahn School of Medicine at Mount Sinai (ISMMS) between 7:30 and 8:00am after an overnight fast. Vital signs, weight, height and waist-hip ratio were measured and then approximately 160 cc of whole blood was collected and processed for subsequent assays. Participants received a 0.50 mg tablet of Dexamethasone to ingest at 11:00pm and returned the following morning at 8:00am (post-Dex) for collection of 10cc of blood.

Blood samples were processed and aliquoted for storage into whole blood, serum, plasma, buffy coat or peripheral blood mononuclear cells (PBMCs) depending on the assay (Table S1). PAXgene tubes were used to collect samples for DNA and RNA. Samples were stored, inventoried and frozen at -80° using Freezerworks software (Mountlake Terrace, WA). Samples were shipped in batches on dry ice to each of the respective assaying laboratories, with the following exceptions: one tube of whole blood (unfrozen) was shipped to UCSF for FACS sorting for cell type and activity on the day of blood collection; the IC-50_DEX_ assay was performed on live (unfrozen) PBMCs beginning on the day of collection; blood samples were delivered to ISMMS or JJP VAMC CLIA-certified laboratories for same-day assays of standard clinical labs, including cell counts. Urine toxicology testing was also conducted at the time of blood collection.

Table S2 contains details for all molecular markers, including tube types for each molecular dataset.

Table S2. Summary of blood tubes and samples for molecular assays.

| Markers | Tube Type |
| --- | --- |
| DNA-Genomic Markers | Yellow Top ACD |
| RNA-Gene Expression | PAX-gene (RNA) |
| Endothelial Progenitor Cells | EDTA Lavender Top (LTT) |
| Cytokines and Brain Derived Neurotrophic Factors | SST Red/Gray Top |
| Neurosteroids and Metabolomics | EDTA Lavender Top (LTT) |
| Oxidative stress markers | EDTA Lavender Top (LTT) |
| miRNA, DNA Methylation and Metabolomics | EDTA Lavender Top (LTT) |
| DNA-Methylation | PAX-gene (DNA) |
| Peripheral Blood Mononuclear Cells | EDTA Lavender Top (LTT) |

1. **Molecular Assays**
   1. **Neuroendocrine Assays**
      1. *Cortisol*

Cortisol levels in plasma was assayed using Cortisol ELISA kits from IBL-America (Minneapolis, MN), following the manufacturer’s instructions. Assay sensitivity: 2.5 ng/mL. The intra-assay and inter-assay coefficients of variation for this assay 5.3% and 9.8%, respectively. Two blood samples were assayed for the determination of cortisol before and after DEX administration. Decline of cortisol from Day 1 to Day 2 (i.e., difference score) is conceptualized as a measure of DEX suppression.

- - 1. *Adrenocorticotropic hormone (ACTH)*

ACTH levels in plasma were assayed by using ACTH ELISA kit (ALPCO Diagnostics, Windham NH), following the manufacturer’s instructions. A dose response curve of absorbance unit vs. concentration was generated using results obtained from the calibrators. Concentrations of ACTH present in the controls and patient samples were determined directly from this curve. Assay sensitivity: 0.5 pg/mL. The intra-assay and inter-assay coefficients of variation for this assay 5.7% and 8.0%, respectively. Two blood samples were assayed for the determination of ACTH before and after DEX administration. Decline of ACTH from Day 1 to Day 2 (i.e., difference score) is conceptualized as a measure of DEX suppression.

- - 1. *Lysozyme Activity Assay (Lysozyme IC50-DEX)*

For the lysozyme IC50-DEX assay, mononuclear leukocytes were prepared immediately following the blood drawing procedure. For the preparation of mononuclear leukocytes, platelet-rich plasma was separated by low speed centrifugation. After collecting plasma, the remaining cells were diluted by the sample volume with Hanks’ Balanced Salt Solution (HBSS) and the lymphocytes were isolated by density centrifugation utilizing Ficoll-Paque (GE Healthcare) and washed twice in HBSS according to the method of Boyum ^1^. The final cell pellet was re-suspended in a medium (RPMI-1640) containing 10% fetal calf serum, penicillin, streptomycin, and L-glutamate (Life Technologies, Grand Island, NY) at a density of 1.75-2.00 x 106 cells/ml.

The test for examining the inhibition of lysozyme synthesis and release was carried out in 96-well culture plate in a total volume of .22 mL, modified from ^2^. Lysozyme activity was measured by turbidimetric method using Micrococcus lysodeikticus (Sigma) as the substrate. Micrococcus lysodeikticus was prepared in 0.1 mol/L phosphate buffer, pH 6.3, at a concentration of .05% and homogenized with a tissue grinder equipped with a Teflon pestle (Wheaton, St. Millville, New Jersey) with 3 strokes. 20 µL of supernatant of cell culture was incubated with 150 µL of substrate in a 96-well plate at 37°C for 7–10 min with shaking and then kinetically read by a microplate reader at 450 nm for 20 min. Cells (3.5-4.0 X 105) were incubated with 0, .5, 1, 2.5, 5, 10, 50, and 100 nmol/L of dexamethasone (DEX) (Sigma) at 37°C in a humidified atmosphere with 5% CO2 for 3 days. Each concentration of DEX was incubated in triplicate. After centrifuging the plate, 120 µL of supernatant were removed and pooled from each triplicate well. The standards were prepared using pure lysozyme from chicken egg white (Sigma) dissolved in RPMI-1640 as used for the cell culture. The inhibition curve was drawn as concentration of DEX versus relative activity of lysozyme. Results were expressed as IC50-DEX (nmol/L) based on the concentration of DEX at which 50% of lysozyme activity was inhibited. The intra- and inter-assay coefficients of variation for the measurement of lysozyme activity were 6.9% and 9.8% respectively (Yehuda et al., 2004).

- 1. **Clinical laboratory values**
     1. *CLIA certified lab analysis*

Blood samples were collected CLIA certified labs at the associated hospitals (James J. Peters VA Medical Center and Mount Sinai Hospital) for analysis.

- - 1. *Insulin – previously published in* ^3^

Fasting venous blood samples were obtained by venipuncture between 0800 and 0900 am, following an overnight fast, processed, and stored at −80 °C. Serum insulin was assayed by a CLIA certified lab procedure, using a chemiluminescent immunoassay (Siemens Centaur XP**,** Siemens Healthcare Diagnostics, Tarrytown, New York).

- 1. **Small Molecules**
     1. *F2 Isoprostanes – previously published in* ^4^

Levels of F2-isoprostanes were quantified by the Eicosanoid Laboratory at Vanderbilt University. F2-isoprostanes were extracted and purified with solid phase extraction and thin layer liquid chromatography and then converted to trimethylsilyl ether derivatives and analyzed by gas chromatography–mass spectrometry (GC–MS) as described previously ^5,6^ The CV was 12% for this assay and Lower Limit Of Quantification (LLOQ) was 0.003 ng/ml.

- - 1. *8-OH-dG – previously published in* ^4^

To determine the concentration of 8-OHdG in plasma, 250 μl of ice cold 10% meta-phosphoric acid was added to 200 μl plasma and the sample incubated for 20 min on ice for protein precipitation. After centrifugation at 18,000g for 15 min at +4°C, the supernatant was filtered through a 0.2 μ filter (PGC Scientific, Frederick, MD) and a 10 μl aliquot was injected into the HPLC–MS system. The mobile phase consisted of 7 mM ammonium acetate, 1.5% methanol and 0.1% formic acid. The chromatography was performed using a Dionex HPLC-UV system coupled to an electrospray ionization (ESI) tandem mass spectrometer (Thermo-Finnigan LCQ) with a Phenomenex Gemini column (C18, 150 × 2.0 mm, 3 μm particle size) at a flow rate 0.3 ml/min and HPLC-UV wavelength of 254 nm. To determine a concentration of 8-OHdG in plasma, a standard curve with known concentrations of 8-OHdG was created and software automatic calculation was performed. CV was 6.3% and LLOQ was 0.2 pmol/column.

- - 1. *GSH/GSSG*

Fasting blood samples were collected into EDTA-Vacutainer tubes and immediately chilled on ice before centrifuging at 4000 x g for 10 minutes at 4^o^C. Plasma aliquots were transferred into cryostat tubes and stored at -80°C until extraction and HPLC quantification. To precipitate proteins, 250 μl ice cold 10% meta-phosphoric acid was added, mixed well, and the sample was incubated for an additional 10 minutes on ice. After centrifugation at 18,000 x g for 15 minutes at 4^o^C, the supernatant was filtered through a 0.2 μm nylon membrane filter (PGC Scientific, Frederick, MD) and a 20 μl aliquot was injected into the HPLC system.

For determination of SAM, SAH and free oxidized (GSSG) and reduced (GSH) glutathione, 100 μl of 10% meta-phosphoric acid was added to 200 μl plasma to precipitate protein; the solution was mixed well and incubated on ice for 30 minutes. After centrifugation for 15 minutes at 18,000 g at 4^o^C, supernatants were passed through a 0.2 μm nylon membrane filter and 20 μl was injected into the HPLC system.

The methodological details for HPLC elution and electrochemical detection have been described previously ^7,8^.(18;19). The analyses were performed using HPLC with a Shimadzu solvent delivery system (ESA model 580) and a reverse phase C_18_ column (5 μm; 4.6 x 150 mm, MCM, Inc., Tokyo, Japan) obtained from ESA, Inc. (Chelmsford, MA). A 20 μl aliquot of plasma extract was directly injected onto the column using Beckman Autosampler (model 507E). All plasma metabolites were quantified using a model 5200A Coulochem II and CoulArray electrochemical detection systems (ESA, Inc., Chelmsford, MA) equipped with a dual analytical cell (model 5010), a 4 channel analytical cell (model 6210) and a guard cell (model 5020). The concentrations of plasma metabolites were calculated from peak areas and standard calibration curves using HPLC software.

- - 1. *Glutathione Peroxidase*

Blood for glutathione peroxidase analysis was collected into EDTA-containing tubes (Vacutainer; BD, Franklin Lakes, NJ). The EDTA tube was inverted 8-10 times, and then kept on ice for 5-10 min, spun at 4°C for 10 min at 2500-3000g, and plasma was separated and stored at –80°C until assay. Plasma was assayed for glutathione peroxidase activity in duplicate, using a commercial glutathione peroxidase enzyme assay kit (BioVision, Inc., Milpitas, CA, USA). In this assay, glutathione peroxidase reduces cumene hydroperoxide while oxidizing GSH (reduced glutathione) to GSSG (oxidized glutathione); the generated GSSG was reduced to GSH with consumption of NADPH by glutathione reductase, and the decrease of NADPH, measured at 340 nm, was proportional to glutathione peroxidase activity, assayed over 20 minutes. 5µl of plasma was used per assay, to obtain glutathione peroxidase enzymatic activities that generate NADPH concentrations within the linear range of the standard curve for NADPH (0 – 100 nmol), assayed at 340 nm. Linear regressions of the data were used to assess changes in absorbance at 340 nm (i.e. NADPH concentrations) over time, to quantitate glutathione peroxidase enzyme activities. Positive and reagent (negative) controls were supplied by the vendor and were run with each assay. Vendor-reported intra-assay variability was 5% and inter-assay variability was 15-20%. PTSD positive and negative participant samples were run in the same assays.

- - 1. *Vitamin C – previously published in* ^4^

Vitamin C was measured using an Agilent 1100 Series high performance liquid chromatograph (HPLC) system with a diode array detector as described previously ^9^. Briefly, serum sample was preserved by adding an equal volume of metaphosphoric acid and treated with dithiothreitol. The resulting supernatant was injected into the HPLC systems equipped with a 250 × 4.6 mm Capcell Pak NH2 column (Shiseido, Tokyo, Japan). The column was equilibrated at 40 °C at a flow-rate of 1 ml/min with a mobile phase composed of monobasic potassium phosphate/H2O/acetonitrile. Vitamin C was analyzed using external standards with UV spectrophotometric detection at 243 nm wavelength. Two quality-control samples were injected at the beginning, the end, and after every 10 samples to monitor intra- and inter-day assay accuracy and precision. Recoveries were consistently in excess of 90% and CV was 4.8%. LLOQ was 0.2 nmol/ml.

- 1. **Metabolomics**

The non-targeted metabolic profiling instrumentation employed for this analysis combined three independent platforms: ultrahigh performance liquid chromatography/tandem mass spectrometry (UHPLC/MS/MS^2^) optimized for basic species, UHPLC/MS/MS^2^ optimized for acidic species, and gas chromatography/mass spectrometry (GC/MS). Samples were processed essentially as described previously ^10,11^. For each sample, 100μL was used for analyses. Using an automated liquid handler (Hamilton LabStar, Salt Lake City, UT), protein was precipitated from the plasma or tissue homogenate with methanol that contained four standards to report on extraction efficiency. The resulting supernatant was split into equal aliquots for analysis on the three platforms. Aliquots, dried under nitrogen and vacuum-desiccated, were subsequently either reconstituted in 50μL 0.1% formic acid in water (acidic conditions) or in 50μL 6.5mM ammonium bicarbonate in water, pH 8 (basic conditions) for the two UHPLC/MS/MS^2^ analyses or derivatized to a final volume of 50μL for GC/MS analysis using equal parts bistrimethyl-silyl-trifluoroacetamide and solvent mixture acetonitrile:dichloromethane:cyclohexane (5:4:1) with 5% triethylamine at 60°C for one hour. In addition, three types of controls were analyzed in concert with the experimental samples: aliquots of a well-characterized human plasma pool served as technical replicates throughout the data set, extracted water samples served as process blanks, and a cocktail of standards spiked into every analyzed sample allowed instrument performance monitoring. Experimental samples and controls were randomized across platform run days.

For UHLC/MS/MS^2^ analysis, aliquots were separated using a Waters Acquity UPLC (Waters, Millford, MA) and analyzed using an LTQ mass spectrometer (Thermo Fisher Scientific, Inc., Waltham, MA) which consisted of an electrospray ionization (ESI) source and linear ion-trap (LIT) mass analyzer. The MS instrument scanned 99-1000 *m/z* and alternated between MS and MS^2^ scans using dynamic exclusion with approximately 6 scans per second. Derivatized samples for GC/MS were separated on a 5% phenyldimethyl silicone column with helium as the carrier gas and a temperature ramp from 60°C to 340°C and then analyzed on a Thermo-Finnigan Trace DSQ MS (Thermo Fisher Scientific, Inc.) operated at unit mass resolving power with electron impact ionization and a 50-750 atomic mass unit scan range.

Metabolites were identified by automated comparison of the ion features in the experimental samples to a reference library of chemical standard entries that included retention time, molecular weight (*m/z*), preferred adducts, and in-source fragments as well as associated MS spectra, and were curated by visual inspection for quality control using software developed at Metabolon ^12^.

For statistical analyses and data display purposes, any missing values were assumed to be below the limits of detection and these values were imputed with the compound minimum (minimum value imputation). Statistical analysis of log-transformed data was performed using “R” (http://cran.r-project.org/), which is a freely available, open-source software package. Welch’s t-tests were performed to compare data between experimental groups. Multiple comparisons were accounted for by estimating the false discovery rate (FDR) using q-values ^13^.

- 1. **Cell Aging and mtDNA**
     1. *Mitochondrial DNA – previously published in* ^14^

Blood was drawn in the morning after a night of fasting. Whole blood was collected into 10 ml EDTA Lavender Top (LTT) tubes and granulocytes were isolated and stored at -80°C. Peripheral blood mononuclear cells (PBMCs) were purified whole blood using standard Ficoll gradient centrifugation method. Granulocytes were prepared from the red blood pellets after Ficoll separation of the PBMCs by lysing in three volumes of ACK lysis buffer (Qiagen, cat #158902). The cells were left in ACK lysis buffer at room temperature for 10 min with inversion every 2 min. The cells were spun at 400 g for 10 min in a Sorvall Legend RT tabletop centrifuge at 10°C. The cell pellets were washed twice with 10 ml of cold DPBS (Invitrogen, cat # 14040-133) and spun at 400 g for 10 min at 10°C. After the second wash, the cell pellets were resuspended in 5 ml of DPBS, aliquoted into 5 of 1.5 ml Eppendorf tubes, spun at 7000 rpm for 5 min at 4°C, and were stored at -80°C for batch DNA purification. DNA was purified using QIAamp blood mini kit (cat# 51106) based on the manufacture's manual and quantity were assessed with a nanodrop spectrophotometer. Relative copy number of mtDNA per diploid nuclear genome was determined using a TaqMan multiplex assay by the detection of a 69 bp fragment of the ND1 gene in mtDNA (nucleotides 3485–3553) and an 87 bp fragment of RNase P (TaqMan® Copy Number Reference Assay, human, RNase P, cat# 4403328, Life Technologies) in the nuclear genomic DNA (nDNA). This assay was adapted from previous published methods ^15^. The primer and probe sequences for ND1 are: ND1-forward [5′-CCCTAAAACCCGCCACATCT-3′], ND1-reverse [5′-GAGCGATGGTGAGAGCTAAGGT-3′], ND1-FAM probe [5′ FAM-CCATCACCCTCTACATCACCGCCC-TAMRA-3′]. The reaction contained 12.5 ng of total cellular (nuclear plus mitochondrial) genomic DNA, 100 nM of ND1 probe, 300 nM each of ND1-forward primer and ND1-reverse primer each, 1 × RNase P copy number Reference Assay, 1 × LightCycler® 480 Probe Master (Roche, cat# 04902343001) in a 10 μl reaction. All samples were run in triplicate wells in 384-well plates in a Roche LightCyler 480. PCR condition was 95°C 10 min for 1 cycle; 45 cycles of 95°C 10 s, 60°C 30 s, 72°C 1 s with data acquisition at 72°C. Crossing point (Cp) for each well was derived by the LightCycler 480 program using the second derivation method. Relative copy number per diploid genome (i.e. per cell) was calculated by the following formula: Relative mtDNAcn = POWER{2, (CpND1 − CpRNaseP)} ∗ 2. Inter-assay CV was 3.4% and intra-assay CV was 0.5%.

- - 1. *Telomere Length*

The telomere length measurement assay was adapted from the published original method by Cawthon ^16,17^. The telomere thermal cycling profile consists of:

Cycling for T(telomic) PCR: denature at 96°C for 1 second, anneal/extend at 54°C for 60 seconds, with fluorescence data collection, 30 cycles. Cycling for S (single copy gene) PCR: denature at 95°C for 15 seconds, anneal at 58°C for 1 second, extend at 72°C for 20 seconds, 8 cycles; followed by denature at 96°C for 1 second, anneal at 58°C for 1 second, extend at 72°C for 20 seconds, hold at 83°C for 5 seconds with data collection, 35 cycles.

The primers for the telomere PCR were *tel1b* [5'-CGGTTT(GTTTGG)_5_GTT-3'], used at a final concentration of 100 nM, and *tel2b* [5'-GGCTTG(CCTTAC)_5_CCT-3'], used at a final concentration of 900 nM. The primers for the single-copy gene (human beta-globin) PCR were *hbg1* [5' GCTTCTGACACAACTGTGTTCACTAGC-3'], used at a final concentration of 300 nM, and *hbg2* [5'-CACCAACTTCATCCACGTTCACC-3'], used at a final concentration of 700 nM. The final reaction mix contains 20 mM Tris-HCl, pH 8.4; 50 mM KCl; 200 μM each dNTP; 1% DMSO; 0.4x Syber Green I; 22 ng E. coli DNA per reaction; 0.4 Units of Platinum Taq DNA polymerase (Invitrogen Inc.) per 11 microliter reaction; 0.5-10 ng of genomic DNA. Tubes containing 26, 8.75, 2.9, 0.97, 0.324 and 0.108ng of a reference DNA (from Hela cancer cells) were included in each PCR run so that the quantity of targeted templates in each research sample can be determined relative to the reference DNA sample by the standard curve method. The same reference DNA was used for all PCR runs.

To control for inter-assay variability, eight control DNA samples were included in each run. In each batch, the T/S ratio of each control DNA was divided by the average T/S for the same DNA from 10 runs to get a normalizing factor. This was done for all eight samples and the average normalizing factor for all 8 samples was used to correct the participant DNA samples to get the final T/S ratio. The T/S ratio for each sample were measured twice. When the duplicate T/S value and the initial value vary by more than 7%, the sample was run the third time and the two closest values were reported. Using this method, the CV for telomere length measurement is typically 3-4%.

- - 1. *Telomerase activity assay by gel (gel-TRAP)*

Gel-TRAP assays were performed by the Telomerase Repeat Amplification Protocol (TRAP) using a commercial kit (TRAPeze Telomerase Detection Kit, Millipore) with modifications ^17^. PBMCs were purified from whole blood collected in CPT tubes (BD). Cells were washed with DPBS and stained by Trypan blue, live cells were counted using a hemocytometer. 5X10^5^ - 1X10^6^ cells per sample were pelleted and lysed with 1XCHAPS buffer as directed by the manual for the TRAPeze kit. An extract corresponding to 5000 cells/μl was made and stored at -80°C and assayed in batches. The reaction was carried out according to the TRAPeze kit manual and run on a 8% polyacrylamide-8M urea sequencing gel. The gel was exposed to a phosphorimager plate overnight and scanned on a Typhoon 8600 Imager (GE Healthcare, Piscataway, NJ). The 293T cancer cell line was used as a positive telomerase activity control and standard. Telomerase activity was expressed as equivalent of number of 293T cells. Telomerase activity was quantified using the software ImageQuant 5.2 (GE Healthcare, Piscataway, NJ). Briefly, signals from the product ladders on the gels were added and normalized against the signal from internal control band for the same lane to get the product/internal control value. For each telomerase activity assay reaction, the product/internal value was divided by the product/internal control value from twenty 293T cells and then multiplied by 20 to obtain the final telomerase activity units, defined as 1 unit = the amount of product from one 293T cell/10,000 immune cells. The average intra assay variability of PBMC samples (N=6, assayed in triplicates) was 8% and the inter-assay variability of PBMC samples (N=24, assayed on 2 different days) was 6.7%.

- 1. **Genetics**

Using DNA extracted from blood samples, genome-wide SNP genotyping of 166 participants was conducted by AKESOgen using the Illumina Infinium PsychArray BeadChip. Standard QC was conducted on each batch separately. The following is our standard QC: using PLINK, SNPs that had a call rate < 95%, a minor allele frequency (MAF) < .05 were excluded, as were samples with > 5% missing data. SNVs failing Hardy-Weinberg proportions (p<1x10^-6^ in controls and p<1x10^-10^ in PTSD cases) were excluded. Furthermore, SNVs call rates that differed significantly between cases and controls were also excluded. After checking for consanguinity (using pairwise associations between each individual in the cohort), no participants were removed based on our threshold of second cousins (pi-hat<0.2). Measures were taken to determine population substructure of the samples. We used PLINK to prune the autosomal data in windows of 50 base pairs, removing one SNP from each pair of SNPs with r^2^>0.25 to obtain a set of roughly independent markers (~50,000 SNPs). Given the representation of different ethnicities in the sample, we included the following HAPMAP populations: YRI, CEU, MEX, GHI, JPN, and CHB. Principal-component analysis (PCA) on the pruned data, including the HAPMAP samples, was then performed to infer axes of ancestry and remove outlier participants. Prior to GWAS, we removed the HAPMAP samples. All samples passed QC except for one, which was removed for low genotyping. 1. Cawthon, R.M., *Telomere measurement by quantitative PCR.* Nucleic Acids Res, 2002. **30**(10): p. e47.

2. Lin, J., et al., *Analyses and comparisons of telomerase activity and telomere length in human T and B cells: insights for epidemiology of telomere maintenance.* J Immunol Methods, 2010. **352**(1-2): p. 71-80.

- 1. **miRNA**

The miRNA biomarker candidates associated with PTSD were identified based on plasma miRNA profiling results generated by small RNA sequencing method. The RNA from plasma samples were isolated using miRNeasy micro kit (Qiagen, Valencia, CA). The quantity and quality of RNA were assessed with NanoDrop spectrophotometer (Thermo Scientific, Wilmington, DE) and Agilent Bioanalyzer (Santa Clara, CA). The miRNA sequencing libraries were generated using a modified small RNA sequencing protocol (available at <http://exrna.org/resources/protocols/)>. The main modification was the addition of 4 random nucleotides at the appropriate end of the adapters to reduce ligation associated sequence bias that were known to be associated with commercial small RNA sequencing library construction kits. Individual library concentrations were measured using the NEBNext Library Quant Kit (New England Biolabs, Ipswich MA), adjusted to a final pooled concentration of 2 nM, and sequenced using the NEXTseq 500 DNA sequencer (Illumina, San Diego CA). The sequencing data were processed using sRNAnalyzer – a small RNA mapping tool developed in-house ^18^. To characterize miRNAs in the sample, the raw sequencing reads were first processed to remove adapter sequences, low complexity sequences, such as homopolymer sequences, and reads with less than 15 nucleotides in length. The processed reads were then mapped against all known human miRNA precursors. A mapped read will then be assigned either to one of the corresponding mature miRNAs (-5p or -3p), or to the precursor, based on which part of the miRNA precursor sequence it was aligned with.

- 1. **Proteomics**
     1. *Peptides - Selected Reaction Monitoring*

The peptide biomarker candidates associated with PTSD were identified based on plasma peptide quantification results generated by SRM (selected reaction monitoring) due to its sensitivity and throughput. Before analysis, the top 14 highly abundant blood proteins such as albumin and ferritin were removed by using immuno-affinity column (Agilent, Santa Clara, CA) and proteins in flow-through were then concentrated and digested with trypsin. For SRM, we assessed the concentration of 1,043 selected proteins including 555 human organ specific proteins and 488 proteins that are associated with various psychiatric diseases. The proteotypic peptides that are unique and specific for the targeted proteins (proteins of interest) were selected using information from SRMAtlas ^19^ and synthesized with heavy isotopic lysine (13C615N2) or arginine (13C615N4) at the C-termini. Optimal running conditions, including Q1/Q3 transition pairs, retention time (RT), collision energy (CE), and the proper amount of spike-in were determined for each heavy peptide standard. A refined scheduled LC-SRM-MS method was then built for mass spectrometer runs based on the desired RT and CE. The measurements were performed on a triple-quadrupole mass spectrometer (Agilent 6490). The results were uploaded and analyzed by Skyline software ^20^. Low quality signals were removed through manual inspection of the results. The ratios between the peak areas of light (endogenous) and heavy spiked-in (L/H) peptides were exported and used for concentration measurement.

- - 1. *Multiplex protein assay*

The Human Neurodegenerative Disease Panel 1 was used to assay Human PTSD plasma samples using Luminex xMAP technology according to the manufacturer’s protocol (Millipore Sigma, Billerica Massachusetts). Briefly, plasma was diluted 1:40,000, and 25 μl was used per sample in duplicate, along with standards and quality controls. Antibody-immobilized magnetic beads were added to capture analytes, a biotinylated detection antibody was introduced, the reaction was incubated with Streptavidin-PE, and the samples were analyzed on the Bio-Plex 200 System (Bio-Rad Laboratories, Hercules, CA) for the analytes of α2-Macroglobulin, Apo Al, Apo CIII, Apo E, Complement C3 and Complement Factor H for each sample.

- - 1. *BDNF*

Blood for BDNF analysis was collected into serum separator tubes (Vacutainer; BD, Franklin Lakes, NJ). After sitting at room temperature for one hour to allow clotting, blood was centrifuged at 2000 x g for 20 min, and serum was separated and stored at –80°C until assay. Serum was assayed for BDNF in duplicate, using a commercial BDNF ELISA assay kit (R&D Systems, Minneapolis, MN, USA)*.* Sera were diluted 1:60 with diluent supplied by the kit manufacturer, to obtain BDNF concentrations within the linear range of the standard curve. To evaluate inter-assay variability, an internal control consisting of serum obtained from a single individual, frozen in multiple aliquots, was run on each plate processed. BDNF concentrations of this control sample were measured on several different days and multiple 96-well plates. The R&D Systems Human BDNF Quantikine ELISA Kit was found to have an acceptable 8-14% inter-assay variability of this control sample, when measured on each plate run with human PTSD positive and negative participant samples. Samples were re-assayed if intra-assay CVs were >10%. PTSD positive and negative participant samples were run in the same assay batch.

- 1. **DNA Methylation**

Genomic DNA was isolated from samples that were collected in PAXgene DNA Blood tubes using the PAXgene Blood DNA kit (Qiagen, Valencia, CA) following ISB’s standard operating procedures (SOP) (ISB-07- 14 SOP). DNA quantity and quality was assessed by Nanodrop 2000 Spectrophotometer (Thermo Scientific, Wilmington, DE) (ISB-07-07 SOP), Qubit® Flourometer (Invitrogen, Carlsbad, CA) (ISB-04-13 SOP) and Agilent Tapestation (Agilent Technologies, CA) (ISB-07-08 SOP).

- - 1. *DNA 450K Methylation Beadchip analysis*

For each sample 500 ng of genomic DNA was bisulfite converted, using the Zymo EZ-96 DNA methylation kit (Zymo Research, CA) following ISB SOP (ISB-07-06 SOP). Samples were randomized across and within plates to minimize potential confounding effects introduced by batch. Genome-wide DNA methylation was carried out using the Illumina Infinium HumanMethylation450K BeadChip arrays (Illumina Inc, CA) according to ISB SOPs (ISB-07-15, ISB-07-16, ISB-04-18 SOPs). In brief the bisulfite-converted genomic DNA was amplified at 37° for 22 hr, fragmented, purified, and hybridized on an 450K BeadChip at 48° for 18 hr. BeadChips were then washed, single-base extension labeled, and stained with multiple layers of fluorescence.

The BeadChips were scanned using the Illumina iScan system (Illumina Inc, CA) (ISB-04-17 SOP). IDAT files containing the raw intensity signals were generated using Illumina’s iControl software; data was analyzed using Genome Studio to check internal controls (ISB-07-17 SOP), and all further analysis carried out in the R environment. The raw data was preprocessed using the Champ R package. Both quantile normalization and BMIQ normalization were applied to adjust the probe type bias. Low detection probes, quality control failure samples, and outliers were removed. Experimental batches, age, and white blood cell types were designated as confounding factors, and therefore these factors were removed from the normalized data using the batch correction function of the LIMMA R package. Several different annotation resources and statistical and visualization tools were also used to determine functional enrichment including KEGG ^21,22^, Ingenuity Pathway Analysis (IPA) (Ingenuity, CA), and Cytoscape ^23^.

- - 1. *Targeted methylation sequencing*

1 µg of genomic DNA was sent to Zymo Research for targeted methylation. Assays were designed targeting CpG sites in the specified regions of interest (ROI) using primers created with Rosefinch, Zymo Research’s proprietary sodium bisulfite converted DNA-specific primer design tool. All primers were then tested using Real-Time PCR with 1 ng of bisulfite-converted control DNA, in duplicate individual reactions. DNA melt analysis was performed to confirm the presence of a specific PCR product. Following primer validation, samples were bisulfite converted using the EZ DNA Methylation-Lightning^TM^ Kit (Zymo Research, CA) according to the manufacturer’s instructions, then assessed for quantity using Nanodrop (Thermo Scientific, Wilmington, DE). Multiplex and the Fluidigm Access Array™ System (Fluidigm, San Francisco, CA) was performed according the to the manufacturer’s instructions. The resulting amplicons were pooled for harvesting and subsequent barcoding according to the Fluidigm instrument’s guidelines. Samples were subsequently indexed for parallel sequencing according to the Fluidigm instrument guidelines (Fluidigm, San Francisco, CA), then purified using the ZR-96 DNA Clean & Concentrator™ kit (Zymo Research, CA). Indexed sample libraries were then sequenced using a MiSeq V2 300bp Reagent Kit and paired-end sequencing protocol according to the manufacturer’s guidelines (Illumina Inc, CA).

Sequence reads were identified using standard Illumina base-calling software and then analyzed using a Zymo Research proprietary analysis pipeline, aligned back to the reference genome using Bismark (http://www.bioinformatics.babraham.ac.uk/projects/bismark/). Index files were constructed using the bismark_genome_preparation command and the entire reference genome.

1. **Candidate Biomarker Identification**

**Section I – Overview of Consortium Biomarker Identification Approach**

In order to leverage skills and expertise across all team members, many approaches were used to down-select and identify candidate PTSD biomarker panels from all available blood datasets (Table S2). Both single and integrated multi-omics approaches were considered, and all candidate biomarkers were identified solely from the Discovery Cohort.

In total, 50 biomarker panels were identified using many classifier types (random forest, logistic regression, SVM, and tree-based boosting), as well as data-driven, hypothesis-driven, and hybrid approaches. A summary of the types of approaches used is shown in Table S3. The details of each of these biomarker identification strategies are then detailed next (Section II). Overall, 384 candidate biomarkers were contained within these 50 identified biomarker panels. Further down-selection was performed using a cohort of 59 recalled participants from the discovery cohort. Details of the panel refinement/down-selection procedure are detailed in Section III.

Table S3. Summary of biomarker identification approaches. Data-driven approaches used algorithms to identify biomarkers from the discovery datasets. Hypothesis-driven approaches selected specific biomarkers based on literature, additional datasets, or similar diseases.

| Approach | Classifier | Data-driven | Hypothesis-driven | Incorporates biological networks | Single-omic application | Multi-omic application |
| --- | --- | --- | --- | --- | --- | --- |
| Method A | SVM | ✓ |  |  | ✓ | ✓ |
| Method B | SVM | ✓ |  |  | ✓ | ✓ |
| Method C | SVM | ✓ |  |  | ✓ | ✓ |
| Method D | Logistic regression | ✓ | ✓ | ✓ |  | ✓ |
| Method E | Tree-based boosting | ✓ |  |  | ✓ | ✓ |
| Method F | LDA | ✓ |  | ✓ | ✓ | ✓ |
| Method G | Polygenic Risk Score |  | ✓ |  | ✓ |  |
| Method H | Random Forest­ | ✓ |  |  |  | ✓ |
| Method I | Lasso | ✓ | ✓ |  |  | ✓ |

**Section II – Details of biomarker identification methods**

*Method A: Support Vector Machines with Recursive Feature Elimination – identification of a miRNA panel, a peptide panel, and a multi-omic panel*

We applied support vector machine (SVM) with recursive feature elimination (SVM-RFE) algorithm to find an optimal subset of peptides and miRNAs to classify PTSD positive and PTSD negative groups. A 5-fold cross-validation procedure was repeated 100 times to identify optimized features and to obtain an unbiased estimation of classification accuracy. The importance of each feature to the classification was determined based on the selection frequency from 5-fold cross-validations. The features were sorted in order of their frequencies. By increasing the number of features, SVM models were constructed and the average classification accuracies were computed. The optimal feature set was then determined at the highest average classification accuracy of the test set. For proteomics, we identified a panel of 20 peptides. Similarly, the SVM-RFE feature selection algorithm was applied to a miRNA sequencing data to identify an optimal feature set showing maximum classification performance. With 100 times 5-fold cross validation, we identified a panel of 28 miRNAs.

For the multi-omic panel, the 20 peptides and 28 miRNAs identified were combined, the same procedure was applied, and 24 features which include 15 peptides and 9 miRNAs were identified.

*Method B: Support Vector Machines with p-value and fold-change filtering – identification of a miRNA panel, a peptide panel, a metabolomics panel, a clinical lab panel, a methylation array panel, a targeted methylation sequencing panel, a protein panel, and six multi-omic panels*

Some of the Discovery Cohort was initially assayed in two different batches, designated, A and B. Thus, we computed the p-values and fold changes separately for each batch in the Discovery Cohort, as well as the combined Discovery Cohort, denoted by PV_A, PV_B, and PV_Combined, and FC_A, FC_B, FC_Combined. Biomarker panels were identified based on individual filtering criteria for each data type. Following biomarker panel identification, 100 rounds of 5-fold cross-validation were performed using a Linear Kernel Support Vector Machine (LSVM) classifier to estimate performance. Data-type specific filtration criteria are detailed next.

miRNA

A single outlier, 201128_P, was excluded based on PCA analysis. Then, a sequence of filtration criteria were applied. In the full Discovery Cohort, PV_Combined<0.01, and FC_Combined>1.05 or FC_Combined<0.95 cutoffs were imposed, resulting in 138 miRNAs. Next, (PV_A + PV_B)/2<0.01, and FC_A and FC_B in the same direction were required, resulting in 40 miRNAs. Selecting only brain-expressed miRNA resulted in 28 miRNAs. Finally, based on correlation>0.85, remaining miRNAs were divided into 7 groups. We selected the best FC_Combined miRNA for each group. The final miRNA biomarker panel consisted of 7 miRNAs.

SRM Peptides

A single outlier, 201210_N, was excluded based on PCA analysis. Then, the following filtration criteria were applied: (1) mean expression > 1, and (2) PV_Combined<0.05 and FC_Combined>1.05 or FC_Combined<0.95. The final peptide panel consisted of only a single peptide.

Metabolomics

Two filtration steps were applied to generate a metabolomics biomarker panel: (1) PV_Combined<0.05, and FC_Combined>1.2 or FC_Combined<0.8, and (2) PV_A<0.05, PV_B<0.05, and FC_A and FC_B in the same direction. A final biomarker panel of three metabolites was selected.

Clinical Lab

Two filtration steps were applied to identify a clinical lab biomarker panel: (1) PV_Combined<0.05 and |LogFC_Combined|>0.2, and (2) FC_A and FC_B in the same direction. The final clinical lab biomarker panel consisted of four biomarkers.

Illumina Methylation array

Array-based methylation biomarkers were identified using a sequence of filtration criteria. First, PV_Combined<0.05, PV_A<0.05, PV_B<0.05, and |LogFC_Combined|>0.2. Next, FC_A and FC_B must occur in the same direction. Additionally, only methylation probes which match to genes with symbol names were included. Finally, probes in “opensea” regions were excluded. A final biomarker panel of 51 probes was identified.

ELISA Protein

In addition to expression of individual proteins, we included a nonlinear combination of C3/A2M (Complement C3/Alpha 2 macroglobulin) expression. Next, only features with PV_Combined<0.05, and FC_A and FC_B in the same direction were retained. The final biomarker panel contained three features.

Targeted Methylation Sequencing

Four filtration steps were applied to identify sequencing-based methylation biomarkers. First, housekeeping genes were removed. Additionally, only probes with mapped gene symbols were included. Next, only probes with PV_Combined<0.1, |LogFC_Combined|>0.5, and FC_A and FC_B in the same direction were retained. Finally probes in “opensea” regions were excluded. A final biomarker panel of 24 probes were identified as candidate biomarkers. Both probe-level and median-value gene summarization of the 13 genes were used as biomarker panels.

Multi-omic panels

1. Direct pairing

We combined the panels described previously to observe where the prediction power grows using a mixed panel. We tried many combinations and selected the six top performing combinations: (1) targeted methylation and miRNAs, (2) targeted methylation and array-based methylation, (3) targeted methylation and metabolites, (4) targeted methylation, miRNAs, and metabolites, (5) targeted methylation, array-based methylation, miRNAs, and metabolites, and (6) targeted methylation, array-based methylation, miRNAs, metabolites, and pulse.

1. Mixed pairing

In a second approach, all single panels were mixed into a combined set. Then we generated 10 new iterations of 5-fold cross-validation, upon which we constructed 50 classifiers and recorded AUCs and weight vectors of LSVM. The feature with the smallest average square weight was removed recursively until the maximum average AUC was achieved. For each run, the best candidate set was recorded. Then we voted the best of the best candidates out of 500 runs. Features with 200, 300, and 400 hits out of 500 runs were reported as three additional panels.

*Method C: L1-norm Support Vector Machines with Recursive Feature Elimination – identification of an endocrine panel, a metabolomics panel, a methylation array panel, a miRNA panel, a peptide panel, and a multi-omic panel*

The feature selection step was done using recursive feature elimination (RFE) with L1-norm support vector machine (SVM) written in Matlab. Once a final set of features were selected, a linear SVM classifier was trained on a set of randomly selected training samples.

All computation was done on Matlab and R.

Our multi-omics classifier is a ‘late-stage’ integration of the five single-omics panels. More specifically, the model is an ensemble classifier determined based on a linear weighted combination of individual single-omics outputs.

Suppose $f_{i}\left( \cdot\right)'s$ are class membership probabilities or scores (for example, discriminant function of SVM) from individual single-omics classifiers. Also, suppose we have cross-validation performances (AUC’s), $\alpha_{i}$. Then we obtain multi-omics class membership probabilities or scores, $f_{MO}\left( \cdot\right)'s$, as performance-weighted convex combinations.

$$f_{MO}\left( \cdot\right)=\sum_{i} \frac{\alpha_{i}}{\sum_{i} \alpha_{i}}f_{i}\left( \cdot\right)$$

Note that, in addition to performances, weights can be determined from practical considerations of the panels. For example, some markers might be measured more reliably and conveniently than others and can be weighted accordingly. Also, the single-omics classifiers can be seen as special cases where all, but one, of the weights are set to zero. In the current application of the algorithm, equal $\alpha_{i}$’s were used.

*Method D: Biologically-inspired feature combinations with Logistic Regression Classifier – identification of a multi-omic panel*

The metabolomics data obtained were already median normalized, therefore, clinical lab, endocrine and immune datasets were median normalized prior to computational analysis. To determine a robust feature set, a combination of bootstrapping and permutation was used. PTSD status labels were permuted 10,000 times to generate a null distribution of test statistics. Statistical properties of the distributions (mean, median, and variance) were then computed on the full dataset using the generated null distribution. Features with p<0.05, q<0.1 and cohen's d of at least 0.2 were retained for further analysis. Additionally, 10,000 bootstrapped datasets were generated (50 cases and 50 controls were selected for each bootstrapped dataset). Features with p<0.05, q<0.1 and cohen's d>0.2 in at least 50% of the bootstrapping iterations were also retained. The retained features from both bootstrapping and permutation analysis were used to construct relevant biological networks. Network information from KEGG, Reactome, Panther, NCI-PID, and literature were used to generate biological networks. Additional features were included when contained in the relevant networks. Based on network structure, ratios were constructed to quantify relevant network biology and physiology. The most statistically significant ratios were used for disease classification. The data were log-transformed for classification, and a logistic regression classifier with Ridge regularization was used to evaluate performance. All classification was performed in Matlab using 'fitclinear'.

*Method E: Gradient Boosted Machines with DEG and DEVG filtering – identification of an endocrine panel, two metabolomics panels, a miRNA panel, a peptide panel, and a multi-omic panel*

Monte Carlo cross-validation was used to generate 100 random partitions of training and testing data subsets of the Discovery Cohort (2/3 training, 1/3 testing). Feature selection was performed on larger datasets (number of features>2000) using a combination of differential expression and differential expression variance analysis. Differential expression analysis identifies features based on differences in group means, while differential expression variance analysis identifies features based on differences in group variances. The top 1000 features from differential expression analysis and top 1000 features from differential expression variance analysis (based on p-value) were retained for model building. The 'caret' and 'gbm' R package were used to build and evaluate models. For each iteration, model parameters were tuned using 3-fold cross-validation on only the training data subset. The model parameters used searched were:, shrinkage=0.001, interaction.depth={1,3,5,8}, n.trees={10,50,100,250,500}, n.minobsinnode=10% of total training participants.

To reduce the size of the biomarker panel, the top features with feature importance of at least 10% of the most important feature, or the top 25 features (ranked by feature importance) were selected for final model building.

*Method F: COMBINER – identification of a metabolomics panel, a methylation array panel, a miRNA panel, a peptide panel, and a multi-omic panel*

COMBINER ^24^ is a tool to discover biomarker markers from omics data in a robust manner. This algorithm takes high-throughput training data as input and produces a panel of candidate biomarkers along with a decision boundary as output. We extend the capabilities of the tool in this paper by incorporating – 1) a filtering step to reduce model complexity and 2) a new strategy in the present framework to perform the multi-omics data integration. The Discovery Cohort set is randomly divided into three equal cohorts (A, B, and C). Each cohort has all the features and randomly chosen participants.

The original COMBINER algorithm ^25^ performs two steps---inference and recursive feature elimination (RFE). In the inference step, data from Cohort A is projected separately onto known biological pathways (from KEGG, PID and REACTOME) and top 100 pathways (based on “pathway activity” scores) along with their “driver genes” are identified. In the RFE step, data from Cohort B corresponding to the top 100 pathways (found in step 1) are used along with linear discriminant analysis (LDA) to identify candidate biomarker pathways and genes. Restricting ourselves to these candidate biomarker pathways, various classification performance metrics are estimated on Cohort C by employing LDA as the linear classifier for cross-validation. The above three-fold partition is repeated 30 times, and the mean values of the performance metrics are used to give the average predictive performance. A majority voting strategy is employed to obtain the final set of robust candidate pathways, selecting only those pathways that occur in at least 15 of 30 splits.

Next, we perform an additional filtering step to reduce the complexity of the obtained statistical model, to prevent over-fitting. In this step, we search all of the driver features in the selected candidate pathways to identity a subset of biomarker features. All the driver features are sorted in the descending order by their absolute t-scores from the entire discovery dataset. As the t-score threshold was decreased, LDA classifiers were trained and tested over 250 rounds of 10-fold cross-validation. The final biomarker set was selected based on the minimum average error rate. This approach was applied to the metabolomics, array-based methylation, miRNA, and peptide datasets to identify four single-omic biomarker panels.

Multi-omic COMBINER

For a given pair of datasets, we consider only participants for which data are available for both modalities. We consider a feature from a given modality to have an effect on a pathway if it is either present in the pathway or has at least one gene with which it is known to interact. Only those pathways which are affected by both modalities are considered. Metabolites were mapped to the pathways using KEGG. miRTarBase database was used to identify high confidence, experimentally verified miRNA targets for mapping to KEGG pathways. The multi-omics data integration version of COMBINER differs in the manner by which driver genes in a given pathway are identified. We consider all pairs of heterogeneous features for a given pathway. In other words, for each pair, one feature must be from modality 1 and the second from modality 2. An average of the expression values is assigned to each of these pairs. As before, the CORG method ^26^ (a greedy search algorithm) is employed on all pair feature expression values to identify the “driver pair of heterogeneous features”. We then run COMBINER to obtain important pathways and subsequently the driver bi-modal features. These steps can be performed for every pair of modalities.

In the final step of data integration, all candidate biomarkers obtained from single-omic and multi-omic analyses are collected. This set of important heterogeneous candidate markers single-omic features and pairs of multi-omic features. The filtering step used above is then employed on the complete heterogeneous dataset to obtain a single multi-omic biomarker panel and to obtain the corresponding decision boundary.

*Method G: Polygenic Risk Score – identification of GWAS panel*

All samples were genotyped with Ilumina’s Infinium PsychArray BeadChip, which contains 303,378 SNP’s. Strand orientation of each chromosome was checked and corrected separately with PLINK. Then pre-phasing was performed with SHAPEIT (https://mathgen.stats.ox.ac.uk/genetics_software/shapeit/shapeit.html) using genetic map data for build 37 (http://www.shapeit.fr/files/). IMPUTE2 (https://mathgen.stats.ox.ac.uk/impute/impute_v2.html) was used for imputation after splitting the chromosome genotypes into a window of 5Mb. 1000 Genome Project phase 3 dataset was used as a reference. Reassembling of the imputed data was done with GTOOL (http://www.well.ox.ac.uk/~cfreeman/software/gwas/gtool.html).

QC filtering was done as follows with PLINK. Variants that do not meet the following criteria were removed: minor allele frequency of at least 0.01, missingness rate of at most 0.1, and Hardy-Weinberg equilibrium p-value of at most 1e-3. This resulted in 9,831,409 variants. Polygenic risk scores (PRS) were built with PRSice ^27^, which implements the standard LD clumping and p-value thresholding approach. The imputed genotype datasets was used as the target dataset and GWAS summary statistics were obtained from PTSD psychiatric genomic consortium study ^28^.

*Method H: Random Forest multi-omic classification – identification of six multi-omic panels*

Six multi-omic panels were identified using the following approach for combinations of four data types. Features were added step-wise, until a maximum AUC was reached. AUC values were computed based on 10-fold cross-validation using a random forest classifier implemented with the caret::train function in R. The six implementations consisted of the following variable combinations: 1) endocrine and biometric datasets, 2) endocrine, biometrics, and GR methylation datasets, 3) endocrine and clinical lab datasets, 4) endocrine, clinical lab, and GR methylation datasets, 5) endocrine, biometrics, and clinical lab datasets, and 6) endocrine, biometrics, clinical lab, and GR methylation datasets.

*Method I: Lasso model – identification of a multi-omic panel*

A Lasso procedure was applied to a subset of metabolomics discovery datasets, identified based on literature and prior knowledge. A five-fold cross-validation procedure was applied 100 times to the Discovery Cohort datasets to estimate performance. All processes, including feature selection and model building, were obtained within cross-validation. The final lasso model was built using the entire training dataset, and the final model included six variables.

*Combining Biomarkers*

A summary of the combined set of candidate biomarkers identified using the methods from Section II is shown in Table S4. In total, 343 candidate biomarkers were identified through the methods detailed in Section II. An additional refinement approach to reduce the size of this candidate biomarker set is detailed in Section III.

Table S4. Summary of candidate biomarkers

| **Data Type** | **Number of candidate biomarkers** |
| --- | --- |
| Biometrics | 2 |
| Clinical Lab | 20 |
| Metabolomics | 27 |
| Methylation – Illumina array | 123 |
| Methylation – targeted sequencing | 33 |
| miRNA – plasma sequencing | 81 |
| Endocrine | 8 |
| Peptide – SRM | 38 |
| Protein – multiplex | 2 |
| Protein – bdnf | 1 |
| Small Molecule – Oxidation Byproducts | 4 |
| Nonlinear feature combinations | 4 |
|  |  |
| **Total** | **343** |

**Section III – Further biomarker down-selection and panel refinement**

To filter and refine the pool of candidate biomarkers, a second cohort (Recall Cohort) was used to refine/filter panels. Many recalled participants experienced symptom changes over the 3.3±0.9 years between the initial and follow-up evaluation.

The pool of candidate biomarkers was refined using the following methodology. Biomarkers were eliminated one-by-one based on biomarker performance. Specifically, 10 iterations of model training and validation were performed for each feature set, with a single biomarker removed. Bootstrapped versions of the discovery and recall cohort were used for model training and validation, respectively. Validation performance was determined based on AUC.

Candidate biomarkers were removed one-by-one based on largest average AUC of remaining biomarker set. After elimination reaches a single remaining biomarker, the biomarker set with the largest average AUC prior to the final performance decline was selected (Fig. S1A). This panel was further refine to filter biomarkers based on Random Forest Variable Importance, keeping only those markers with at least 30% of the maximum importance score (Fig. S1B).


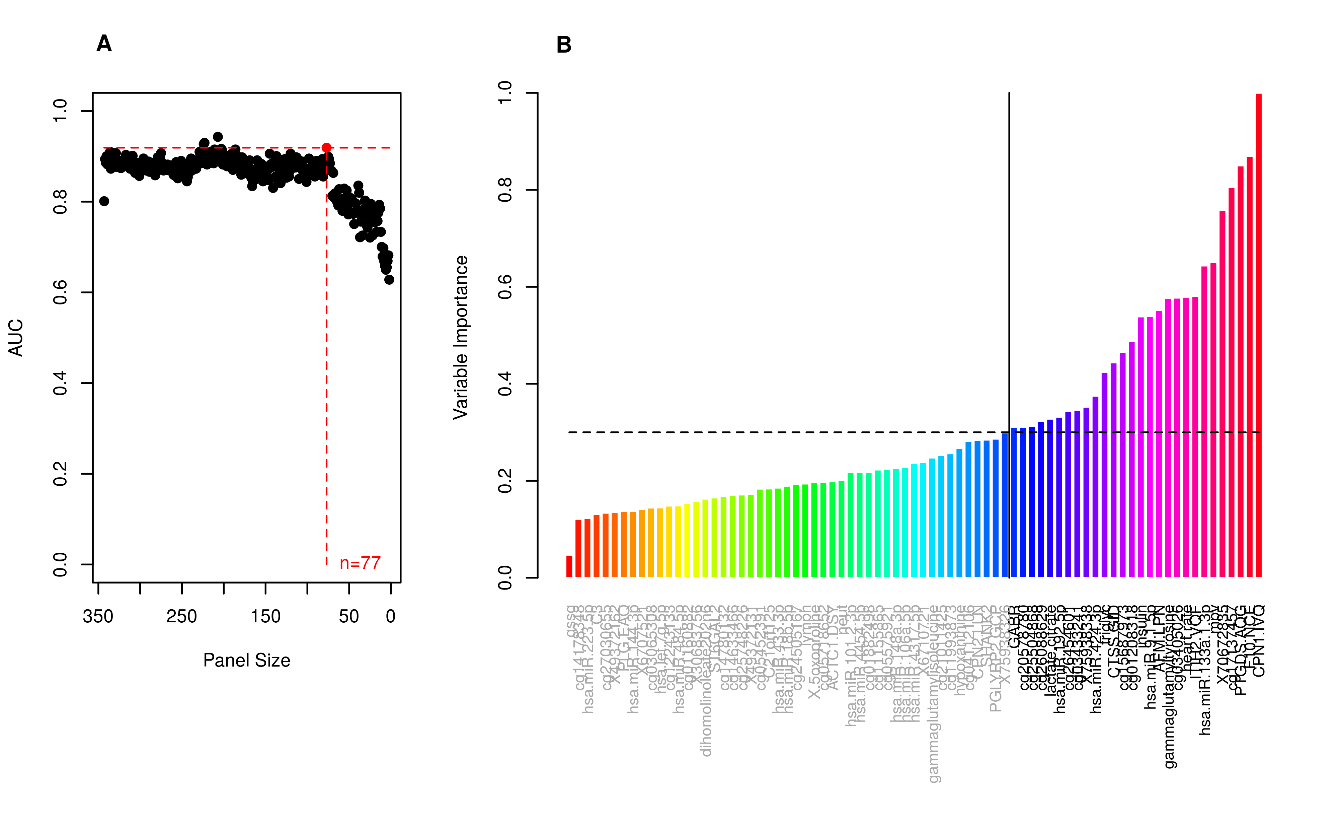


Fig. S1. Two stage biomarker down-selection approach. **(A)** Stage 1: one-by-one feature elimination using recalled participants for biomarker validation. Features are eliminated one at a time, starting with n=343, based on maximum average validation AUC. The selected local maximum validation AUC occurs at n=77 biomarkers. **(B)** Stage 2: random forest variable importance ranking. Remaining 77 biomarkers were sorted based on random forest variable importance in Cohort 2. Features with importance >30% of maximum importance are retained for final biomarker validation (n=28).


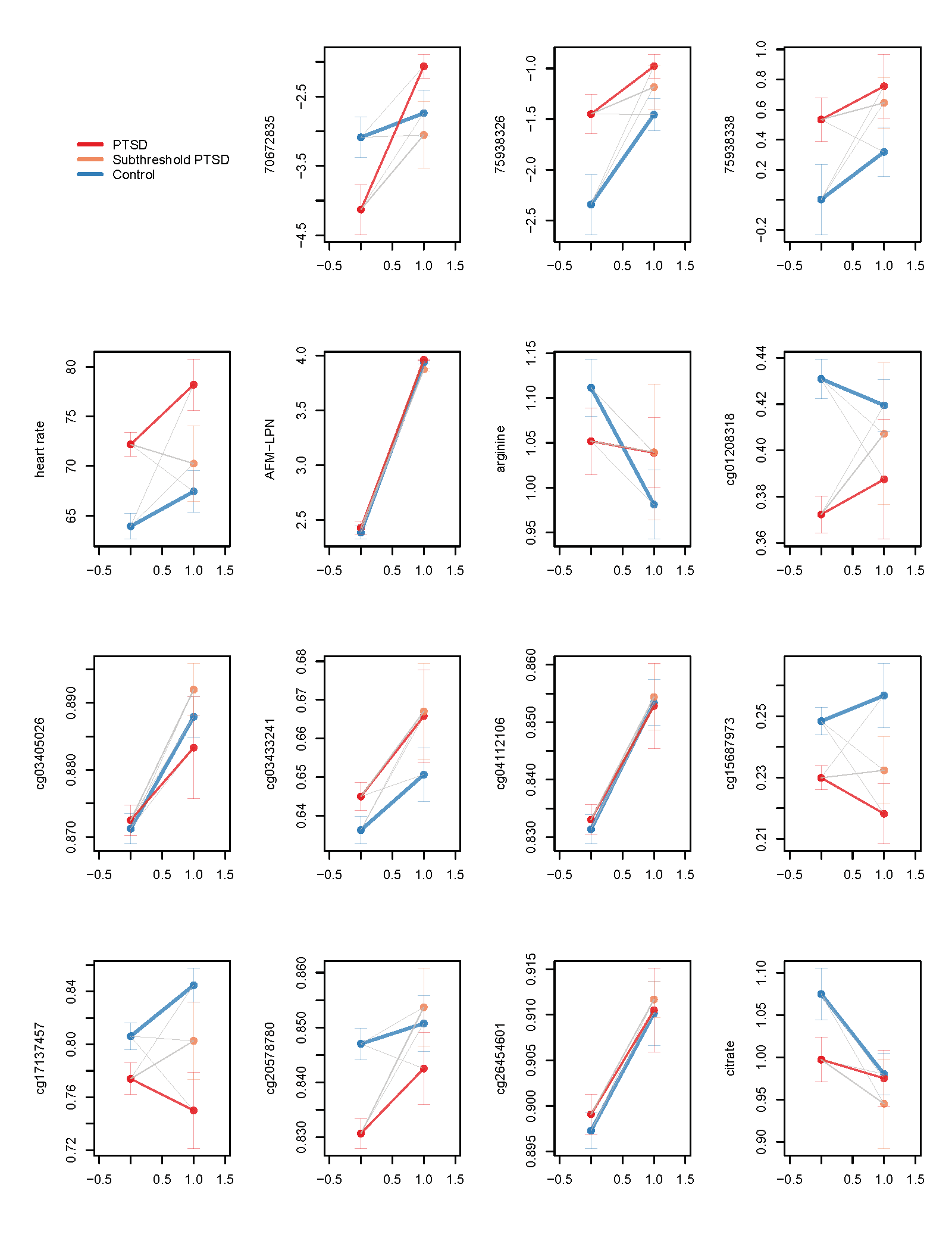

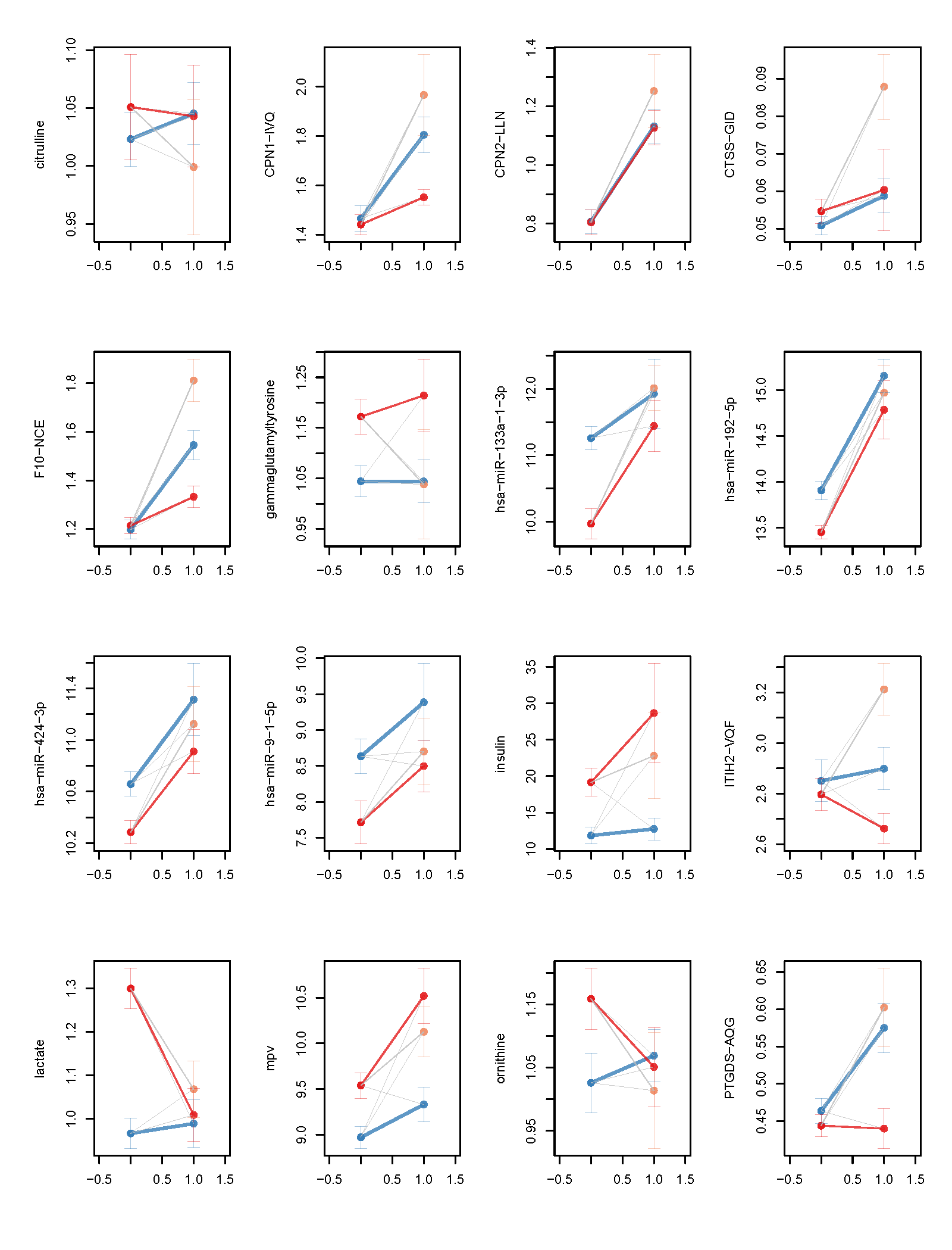


Fig. S2. Biomarker changes in recalled participants. T0 and T1 group means are shown for PTSD (red), subthreshold PTSD (light red), and control participants (blue). Error bars indicate standard error.

**Section IV – Validation of final biomarker panel in independent dataset**

To validate the feasibility and performance of the final identified biomarker panel, an additional male veteran cohort was recruited to form the validation cohort. The final biomarker panel was trained using the combined Cohorts 1 and 2, and validated in the validation cohort (Cohort 3).


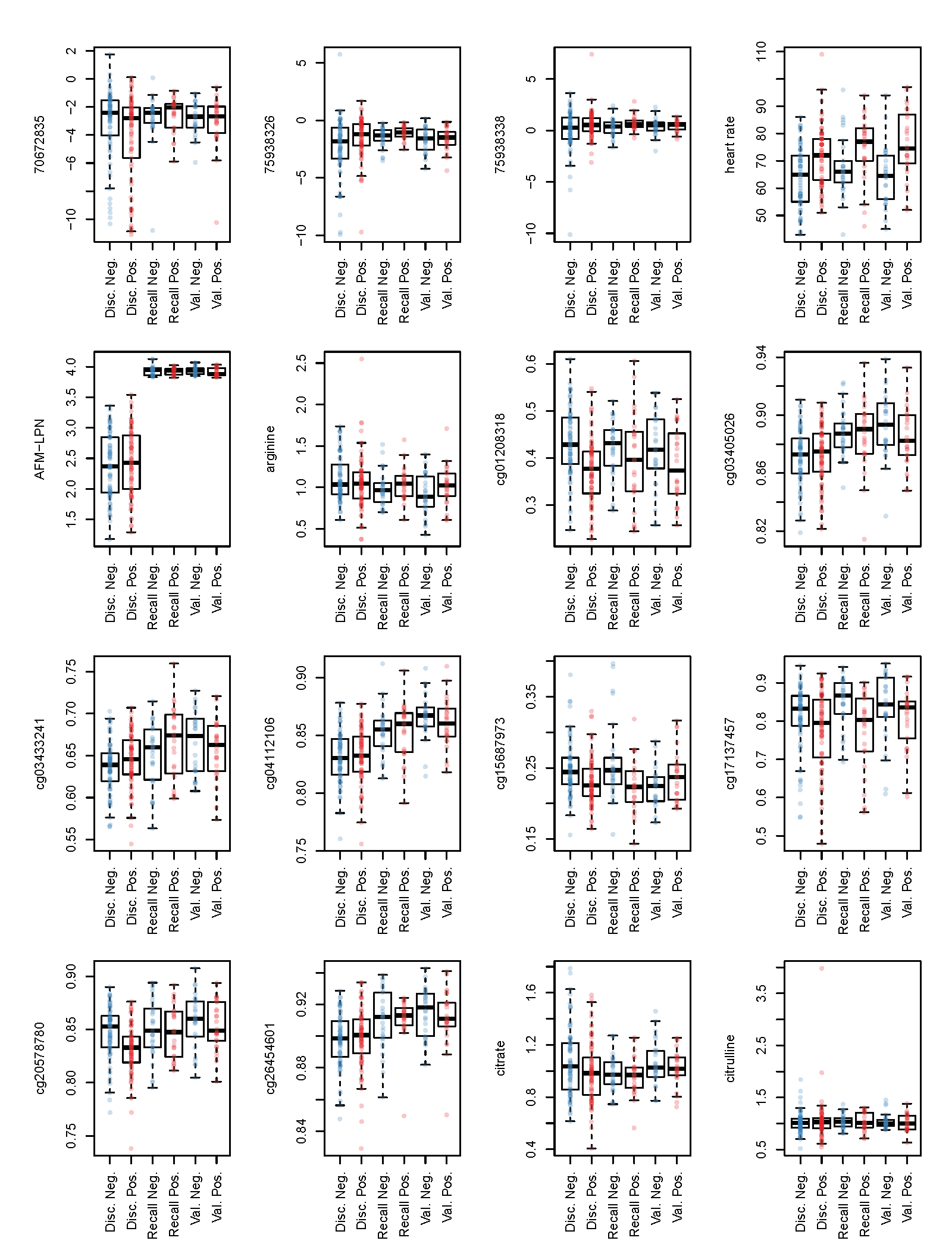


**
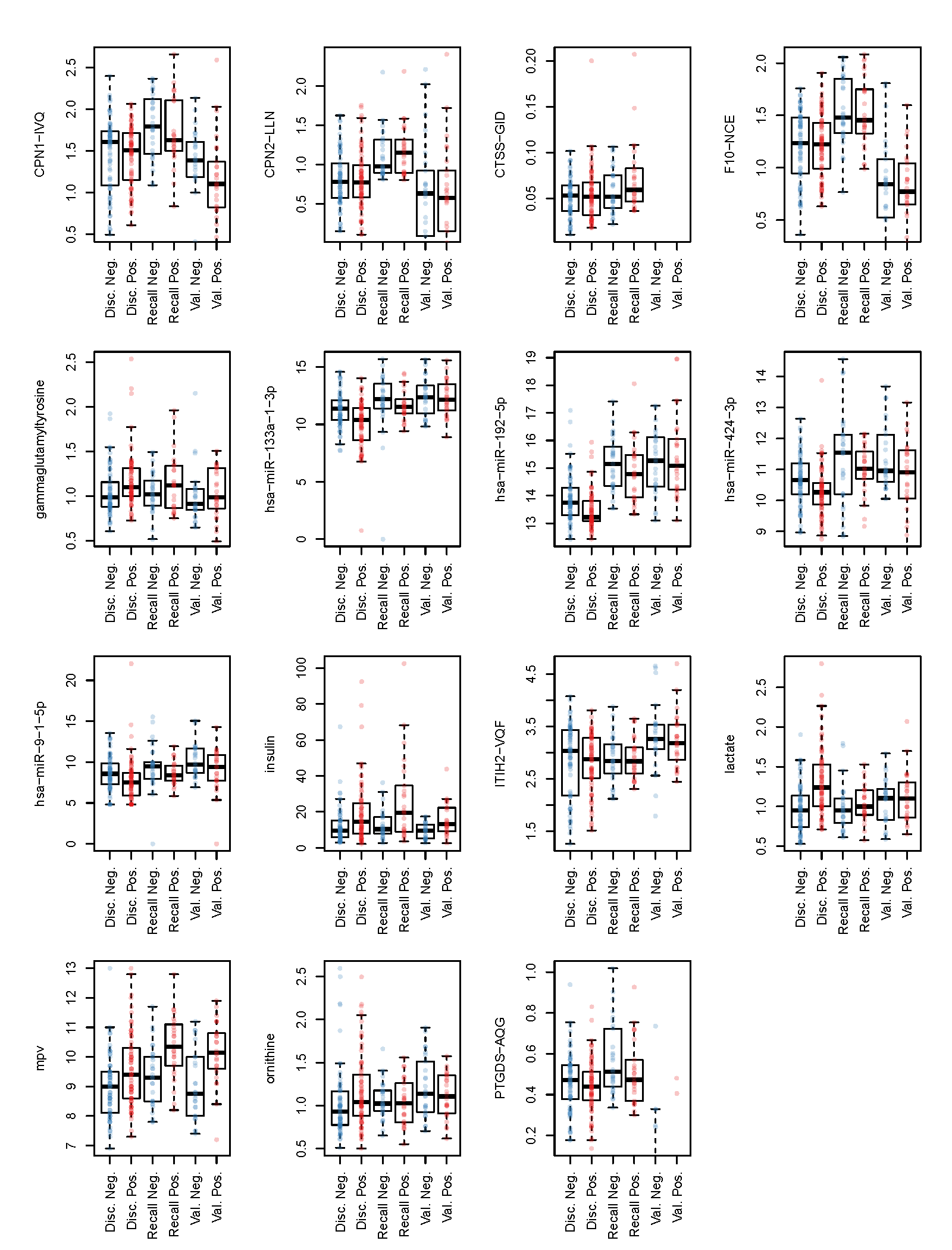
**

Fig. S3. Distribution of final biomarkers in Discovery, Recall, and Validation cohorts.


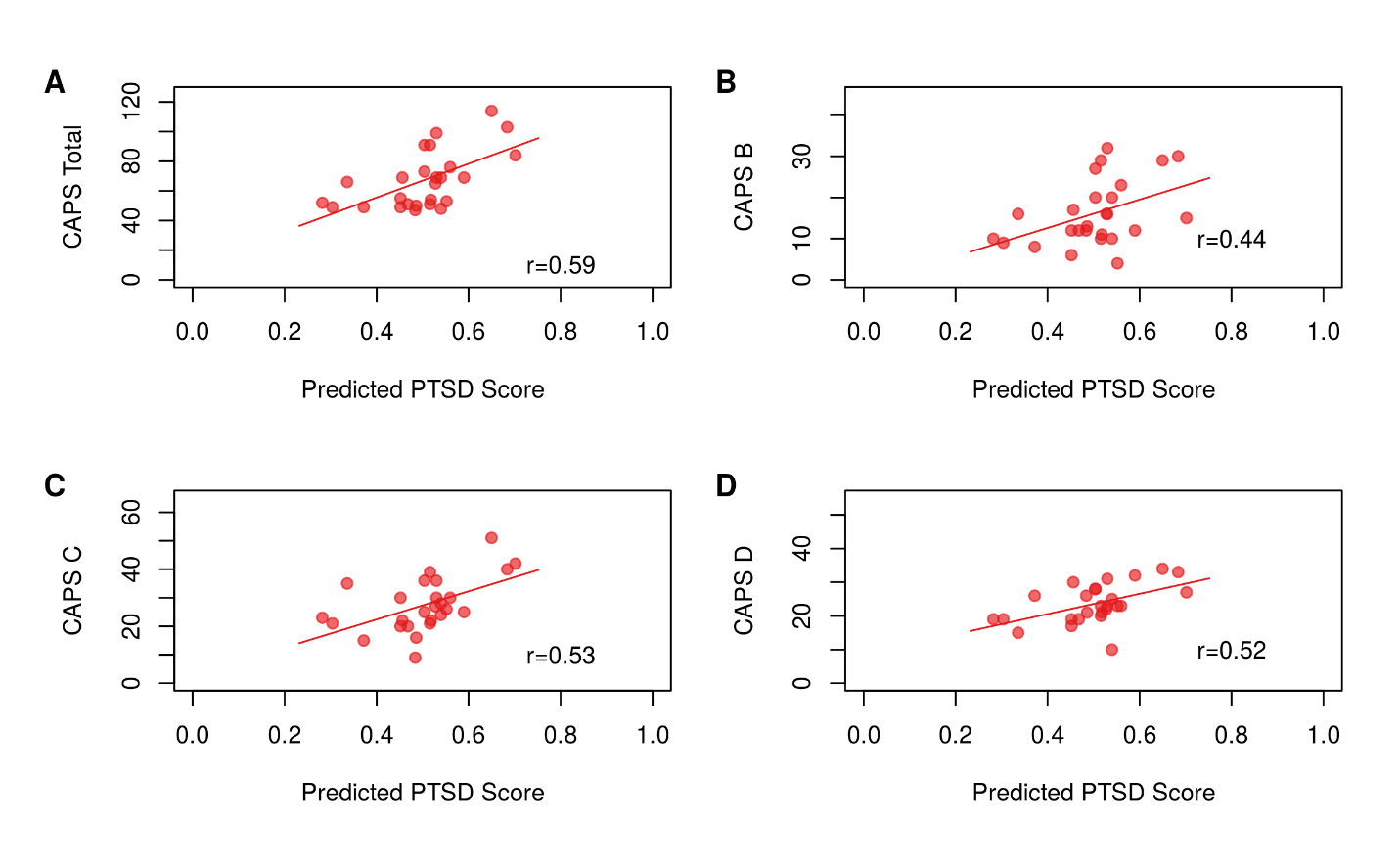


Fig. S4. Predicted probability of PTSD based on trained random forest model using a biomarker panel of 28 features. **(A)** In PTSD participants, predicted PTSD probability is correlated with PTSD symptom severity, measured by total CAPS (r=0.59, p<0.01). **(B)-(D)** Individual CAPS sub-scores (B, C, and D) are also moderately correlated predicted PTSD probability (r=0.44-0.53).


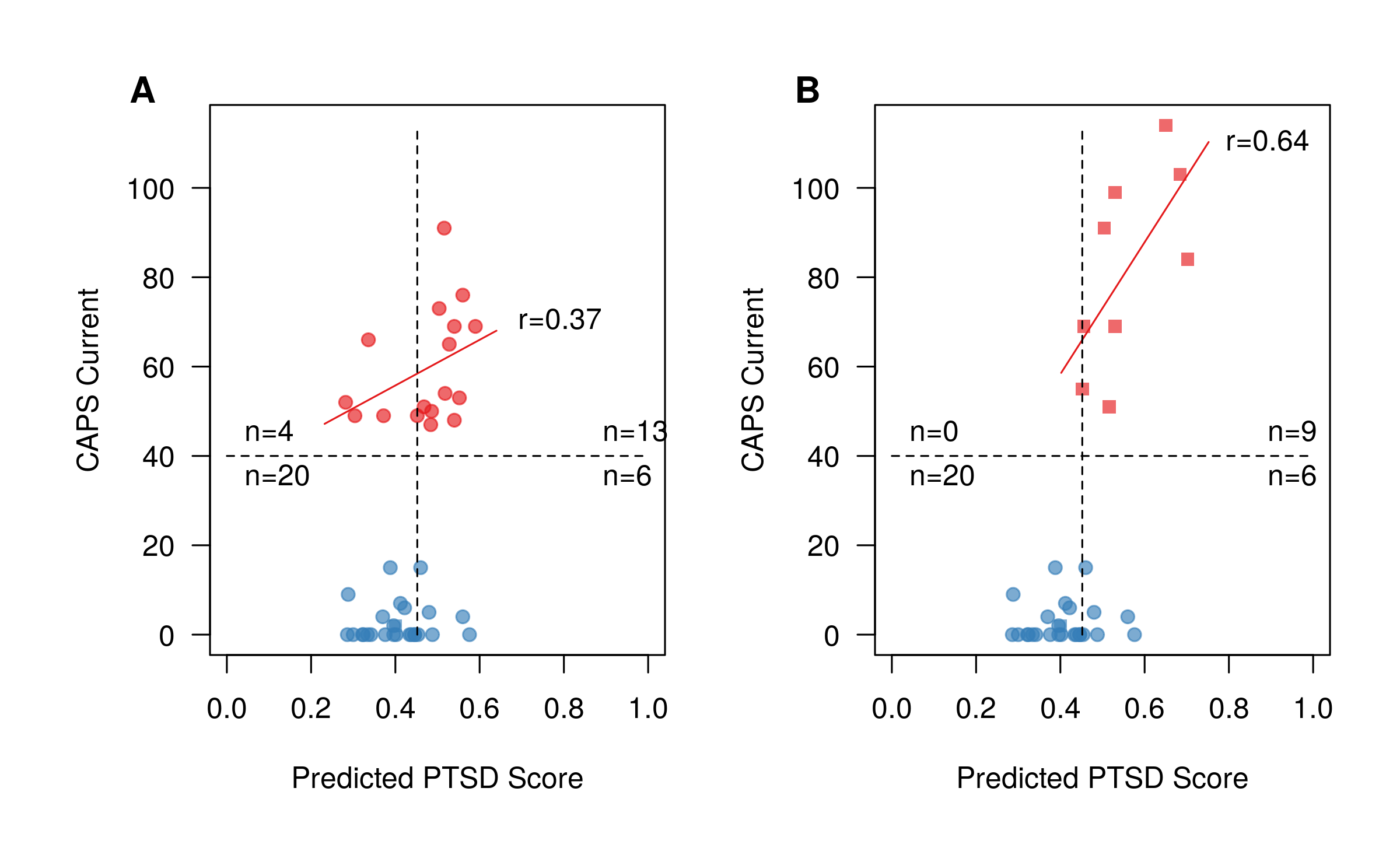
Fig. S5. Predicted probability of PTSD based on trained random forest model using a biomarker panel of 28 features in validation cohort (26 PTSD, 26 control). **(A)** In PTSD+/MDD- participants, predicted PTSD probability is only weakly correlated with PTSD symptom severity, measured by CAPS (r=0.37). **(B)** In PTSD+/MDD+ participants, predicted PTSD probability is more strongly correlated with PTSD symptom severity, measured by CAPS (r=0.64).

**References**

1 Boyum A. Isolation of leucocytes from human blood. A two-phase system for removal of red cells with methylcellulose as erythrocyte-aggregating agent. *Scand J Clin Lab Invest Suppl* 1968; **97**: 9–29.

2 Panarelli M, Holloway CD, Mulatero P, Fraser R, Kenyon CJ. Inhibition of lysozyme synthesis by dexamethasone in human mononuclear leukocytes: an index of glucocorticoid sensitivity. *J Clin Endocrinol Metab* 1994; **78**: 872–877.

3 Blessing EM, Reus V, Mellon SH, Wolkowitz OM, Flory JD, Bierer L *et al.* Biological predictors of insulin resistance associated with posttraumatic stress disorder in young military veterans. *Psychoneuroendocrinology* 2017; **82**: 91–97.

4 Lindqvist D, Dhabhar FS, James SJ, Hough CM, Jain FA, Bersani FS *et al.* Oxidative stress, inflammation and treatment response in major depression. *Psychoneuroendocrinology* 2017; **76**: 197–205.

5 Milne GL, Yin H, Brooks JD, Sanchez S, Jackson Roberts L, Morrow JD. Quantification of F2-Isoprostanes in Biological Fluids and Tissues as a Measure of Oxidant Stress. *Methods Enzymol* 2007; **433**: 113–126.

6 Morrow JD, Roberts II LJ. Mass Spectrometric Quantification of F2-Isoprostanes in Biological Fluids and Tissues as Measure of Oxidant Stress. *Methods Enzymol* 1999; **300**: 3–12.

7 Melnyk S, Pogribna M, Pogribny I, Hine RJ, James SJ. A new HPLC method for the simultaneous determination of oxidized and reduced plasma aminothiols using coulometric electrochemical detection. *J Nutr Biochem* 1999; **10**: 490–497.

8 Melnyk S, Pogribna M, Pogribny IP, Yi P, James SJ. Measurement of plasma and intracellular S-adnosylmethionine and S-adenosylhomocysteine utilizing coulometric electrochemical detection: Alterations with plasma homocysteine and pyridoxal-5’-phosphate concentrations. *Clin Chem* 2000; **46**: 265–272.

9 Margolis SA, Schapira RM. Liquid chromatographic measurement of L-ascorbic acid and D-ascorbic acid in biological samples. *J Chromatogr B Biomed Appl* 1997; **690**: 25–33.

10 Evans AM, DeHaven CD, Barrett T, Mitchell M, Milgram E. Integrated, Nontargeted Ultrahigh Performance Liquid Chromatography/Electrospray Ionization Tandem Mass Spectrometry Platform for the Identification and Relative Quantification of the Small-Molecule Complement of Biological Systems. *Anal Chem* 2009; **81**: 6656–6667.

11 Ohta T, Masutomi N, Tsutsui N, Sakairi T, Mitchell M, Milburn M V. *et al.* Untargeted Metabolomic Profiling as an Evaluative Tool of Fenofibrate-Induced Toxicology in Fischer 344 Male Rats. *Toxicol Pathol* 2009; **37**: 521–535.

12 Dehaven CD, Evans AM, Lawton KA. Organization of GC / MS and LC / MS Metabolomics Data into Chemical Libraries. *J Cheminform* 2010; **2**.

13 Storey JD, Tibshirani R. Statistical significance for genomewide studies. *Proc Natl Acad Sci* 2003; **100**: 9440–9445.

14 Bersani FS, Morley C, Lindqvist D, Epel ES, Picard M, Yehuda R *et al.* Mitochondrial DNA copy number is reduced in male combat veterans with PTSD. *Prog Neuro-Psychopharmacology Biol Psychiatry* 2016; **64**: 10–17.

15 He L. Detection and quantification of mitochondrial DNA deletions in individual cells by real-time PCR. *Nucleic Acids Res* 2002; **30**: e68.

16 Cawthon RM. Telomere measurement by quantitative PCR. *Nucleic Acids Res* 2002; **30**: e47.

17 Lin J, Epel E, Cheon J, Kroenke C, Sinclair E, Bigos M *et al.* Analyses and comparisons of telomerase activity and telomere length in human T and B cells: Insights for epidemiology of telomere maintenance. *J Immunol Methods* 2010; **352**: 71–80.

18 Wu X, Kim T-K, Baxter D, Scherler K, Gordon A, Fong O *et al.* sRNAnalyzer—a flexible and customizable small RNA sequencing data analysis pipeline. *Nucleic Acids Res* 2017; **45**: 12140–12151.

19 Kusebauch U, Campbell DS, Deutsch EW, Chu CS, Spicer DA, Brusniak MY *et al.* Human SRMAtlas: A Resource of Targeted Assays to Quantify the Complete Human Proteome. *Cell* 2016; **166**: 766–778.

20 MacLean B, Tomazela DM, Shulman N, Chambers M, Finney GL, Frewen B *et al.* Skyline: An open source document editor for creating and analyzing targeted proteomics experiments. *Bioinformatics* 2010; **26**: 966–968.

21 Kanehisa M, Goto S. KEGG: Kyoto encyclopedia of genes and genomes. *Nucleic Acids Res* 2000; **28**: 27–30.

22 Kanehisa M, Goto S, Sato Y, Furumichi M, Tanabe M. KEGG for integration and interpretation of large-scale molecular data sets. *Nucleic Acids Res* 2012; **40**: D109–D114.

23 Smoot ME, Ono K, Ruscheinski J, Wang PL, Ideker T. Cytoscape 2.8: New features for data integration and network visualization. *Bioinformatics* 2011; **27**: 431–432.

24 Thakur GS, Daigle Jr. BJ, Qian M, Dean KR, Zhang Y, Yang R *et al.* A Multimetric Evaluation of Stratified Random Sampling for Classification: A Case Study. *IEEE Life Sci Lett* 2016; **2**: 43–46.

25 Yang R, Daigle Jr. BJ, Petzold LR, Doyle III FJ. Core module biomarker identification with network exploration for breast cancer metastasis. *BMC Bioinformatics* 2012; **13**. doi:10.1186/1471-2105-13-12.

26 Lee E, Chuang H-Y, Kim J-W, Ideker T, Lee D. Inferring pathway activity toward precise disease classification. *PLoS Comput Biol* 2008; **4**: e1000217.

27 Euesden J, Lewis CM, O’Reilly PF. PRSice: Polygenic Risk Score software. *Bioinformatics* 2015; **31**: 1466–1468.

28 Duncan LE, Ratanatharathorn A, Aiello AE, Almli LM, Amstadter AB, Ashley-Koch AE *et al.* Largest GWAS of PTSD (N=20 070) yields genetic overlap with schizophrenia and sex differences in heritability. *Mol Psychiatry* 2018; **23**: 666–673.
